# Deep learning for blood glucose prediction: Reproducibility challenges and factors affecting differential performance

**DOI:** 10.1371/journal.pdig.0001633

**Published:** 2026-09-03

**Authors:** Baiying Lu, Biratal Wagle, Zhaohui Liang, Yanjun Cui, Temiloluwa Prioleau

**Affiliations:** 1 Department of Computer Science, Dartmouth College, Hanover, New Hampshire, United States of America; 2 Department of Quantitative Biomedical Sciences, Dartmouth College, Hanover, New Hampshire, United States of America; 3 Department of Computer Science, Emory University, Atlanta, GeorgiaUnited States of America; Mayo Clinic Arizona, UNITED STATES OF AMERICA

## Abstract

Blood glucose prediction is a critical component of next-generation diabetes technologies, such as artificial pancreas systems, where reliable performance is essential for safety and effectiveness. Although deep learning methods have achieved promising advances in this area, a critical gap remains in understanding the reproducibility and generalizability of these methods. To contextualize the gap, this study reviewed 67 recent papers that proposed a deep learning method for glucose prediction to identify key reproducibility challenges. Next, we adopted a standardized framework, encompassing technical, statistical, and conceptual reproducibility evaluations, to experimentally assess the reproducibility of eight representative deep learning methods. To achieve this, we reimplemented and evaluated these eight deep learning methods using over 1.36 million continuous glucose monitoring samples (5,061 days) from 128 individuals with type 1 diabetes across three public datasets: OhioT1DM, DiaTrend, and T1DEXI. We found that even though these models demonstrated good technical and statistical reproducibility, their conceptual reproducibility—the ability to generalize to datasets with different diabetes management patterns—was limited. Further analyses revealed that each model’s overall prediction performance was strongly influenced by individual glycemic control, with higher prediction errors observed among participants with lower time with blood glucose in the target range (70–180 mg/dL). This study identified key reproducibility challenges associated with current blood glucose prediction methods within type 1 diabetes populations, highlighting the need for increased transparency, dataset diversity, standardized evaluation practices, and code accessibility to ensure reproducible and reliable models for blood glucose prediction.

## Author summary

Managing type 1 diabetes requires individuals to frequently monitor their blood glucose levels and constantly consider how decisions related to food, activity, insulin use, and more affect their blood glucose levels. Accurate blood glucose prediction is critical to inform diabetes management strategies and mitigate adverse events. Consequently, many researchers have explored deep learning models for predicting future blood glucose levels, with the goal of providing earlier warnings of adverse events and personalized guidance for diabetes management. However, despite the progress, little effort exists on evaluating the reproducibility of published models for blood glucose prediction. To address this gap, we evaluated the reproducibility of eight state-of-the-art deep learning models using three publicly available datasets from distinct populations with type 1 diabetes. This evaluation allowed us to examine not only whether the reported results could be accurately replicated but also whether these models could generalize to new groups of individuals with different blood glucose management strategies and outcomes. Findings from this study revealed several barriers that limit model reproducibility. Furthermore, we found that the performance of many models was significantly affected by the heterogeneity of glucose management across individuals. Our work highlights the importance of transparent research practices and evaluation across diverse datasets to ensure that future glucose prediction models are reliable and equitable for all people living with diabetes.

## Introduction

Diabetes is a leading chronic condition with significant implications and costs in the U.S. and globally [1–3]. At the individual level, the complexity of having diabetes, especially type 1 diabetes, requires daily (and life-long) management of blood glucose (BG) levels to maintain a healthy standard of living and reduce the risk of micro- and macro-vascular complications [4]. Yet, the majority of people with diabetes (e.g., 79% of adults with diabetes and 83% of youths with type 1 diabetes in the U.S.) do not achieve clinical targets or risk factor control goals [3, 5]. To facilitate the complex task of managing diabetes, technological innovations such as continuous glucose monitors (CGMs) and automated insulin delivery (AID) systems are being used to transform the standard of care [6, 7]. However, several advanced technologies rely heavily on the ability to accurately predict blood glucose in advance to support important management decisions that seek to prevent adverse glycemic events (i.e., hypoglycemia and hyperglycemia). Over the past several years, deep learning has been increasingly applied to blood glucose prediction tasks, leading to a growing body of research that explores various model architectures and prediction strategies [8]. However, as diabetes technology continues to advance, a comprehensive evaluation of machine learning methods for blood glucose prediction is critical. Yet, limited research exists on assessing the reproducibility and generalizability of state-of-the-art (SOTA) methods, particularly deep learning [9–11] that are widely used for blood glucose prediction.

**Fig 1:**
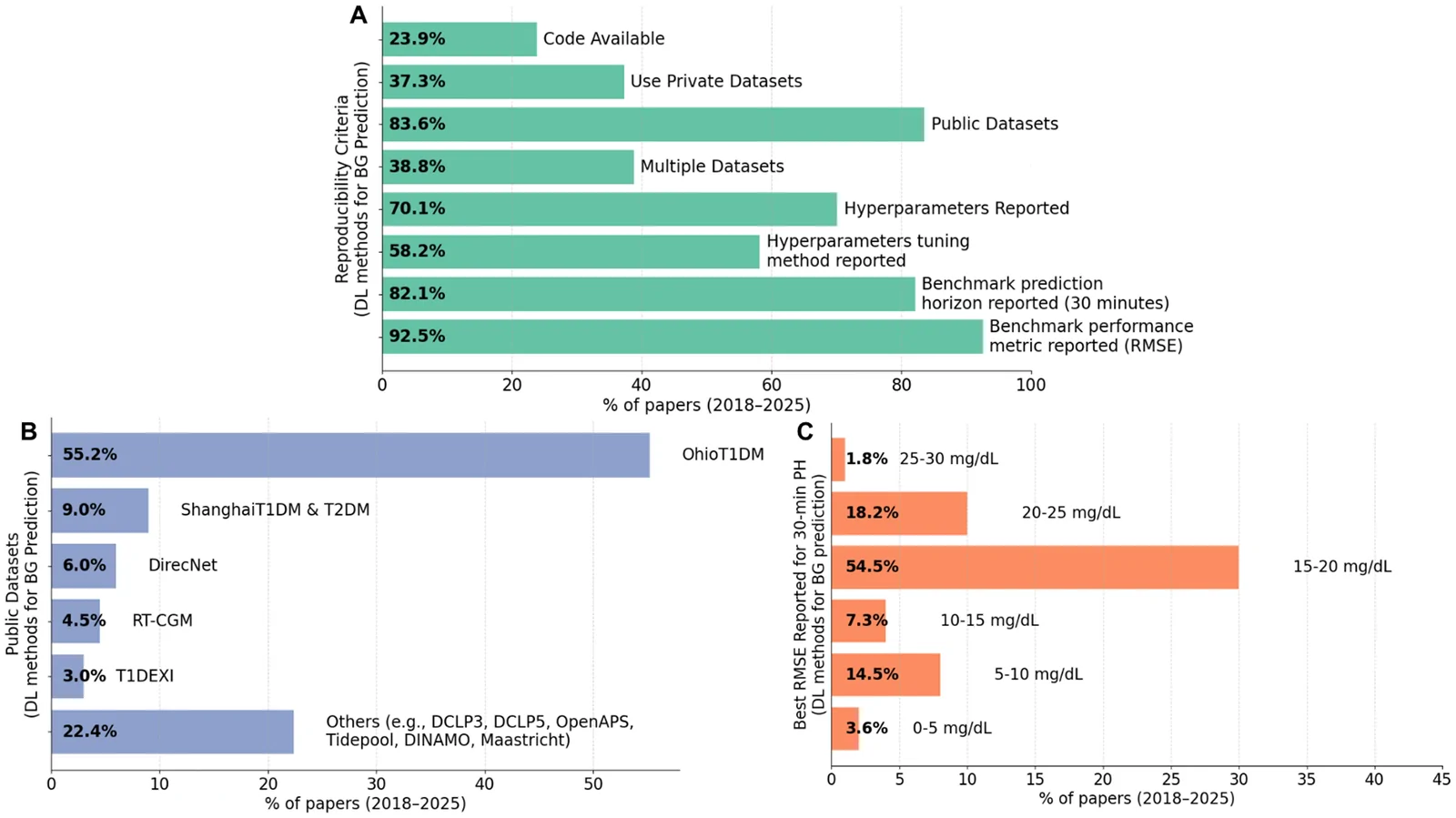
Overview of deep learning papers for blood glucose prediction between 2018 and 2025. **A)** Scoring across 8 reproducibility criteria, **B)** Public datasets used for model development and evaluation, **C)** Best RMSE reported for short-term (i.e., 30-minute) glucose prediction.

To examine potential reproducibility gaps and challenges, we analyzed 67 peer-reviewed papers published between 2018 and 2025 presenting deep learning methods for blood glucose prediction (see supplementary S1 File). This effort revealed substantial limitations in methodological transparency across many published work. In particular, we found that only 23.9% of studies made their code publicly available and 37.3% developed and evaluated their models using private datasets - see Fig 1. These findings suggest that there is limited access to implementation details and training data which is a major barrier for reproducibility studies. Beyond the issue of data and code availability, we also observed an over-reliance on a small number of public datasets. Eighty-five percent (85%) of papers evaluated on at least one public dataset, and the OhioT1DM dataset [12] - with only 12 participants - was the most commonly used public dataset, appearing in 55.2% of studies. This narrow evaluation strategy also has the potential to limit model generalizability and challenge the robustness of reported results. Furthermore, we observed significant variability of reported results across papers. While over 80% of studies reported root mean square error (RMSE) when predicting glucose values 30 minutes in advance, RMSE values varied widely, ranging from below 5 mg/dL to above 25 mg/dL; thereby making it difficult to assess the reliability of reported results. These observations indicate that while deep learning-based methods are promising for blood glucose prediction, reproducibility studies are critically needed in this domain.

In this study, we adopted the framework proposed by McDermott et al. [13] to inform our approach for reproducibility evaluation. McDermott et al. argue that “in order for a study to be fully reproducible, it must meet three criteria: 1) technical reproducibility (can results be reproduced under technically identical conditions?); 2) statistical reproducibility (can results be reproduced under statistically identical conditions?), and 3) conceptual reproducibility or replicability (can results be reproduced under conceptually identical conditions?)” [13]. Guided by this framework, we selected eight representative deep learning studies that evaluated their methods on the publicly available OhioT1DM [12] to assess technical and statistical reproducibility using the same dataset and code (when available). Furthermore, we evaluated conceptual reproducibility using two additional publicly available datasets (DiaTrend [14] and T1DEXI [15]) that feature greater diversity in participants and diabetes management. Our approach sought to conduct a comprehensive assessment of reproducibility under consistent conditions as well as an examination of how each model’s performance responds when applied to datasets with different behavioral or management profiles. By situating reproducibility within a structured evaluation paradigm, this study aims to clarify the extent to which current blood glucose prediction methods generalize beyond their original development settings and to provide insights for developing more robust and clinically meaningful machine-learning approaches for diabetes care.

The key contributions of this work are as follows:

- We systematically evaluated the reproducibility of deep learning–based methods for blood glucose prediction through a review of the literature and standardized experimental replication, providing the first empirical assessment of reproducibility in this domain and identifying key challenges that hinder consistent replication.
- We compared state-of-the-art deep learning methods for blood glucose prediction under a unified experimental setup, standardizing the input datasets and evaluation metrics to assess the adaptability of diverse time-series architectures.
- We identified key factors underlying differential prediction performance by analyzing the relationships among glycemic management, demographic variables, and model prediction. Our findings underscore the importance of expanding data diversity and improving the representation of individuals with suboptimal glucose management in evaluation datasets.

## Materials and methods

This study systematically evaluates the reproducibility of eight state-of-the-art deep learning methods for blood glucose prediction across three publicly available datasets (OhioT1DM [12], DiaTrend [14], and T1DEXI [15]). Following the reproducibility framework proposed by McDermott et al. (2021) [13], we assessed technical, statistical, and conceptual reproducibility through standardized experimental replication. The following sections describe the datasets, selected models, and implementation procedures adopted for our reproducibility evaluation study.

### Ethics statement

This study involves analysis of *only* publicly available de-identified data. As a result, the Committee of Protection of Human Subjects at Dartmouth College determined that the proposed activities are not research involving human subjects, thus institutional review board (IRB) review was not required. The IRB waiver is documented in STUDY00033374.

### Data description

To evaluate the reproducibility of deep learning models across diverse populations and contexts, three publicly available datasets were used, namely **OhioT1DM** [12], **DiaTrend** [14], and **T1DEXI** [15]. These datasets comprise high-quality data collected from FDA-approved CGM devices manufactured by Dexcom, Abbott, and Medtronic [16]. Our selected datasets also differ substantially in cohort characteristics, data collection protocols, and glycemic management profiles. Table 1 presents an overview of three clinically validated metrics for assessing CGM data, namely average blood glucose, time in range (TIR), and glycemic variability (GV) [17]. The average daily blood glucose reflects the mean of daily blood glucose levels. Time in Range (TIR) is an indicator of glycemic control within the target euglycemic range of 70–180 mg/dL and has been associated with a reduced risk of complications [18]. Glycemic variability (GV) captures the degree of glucose fluctuations and is linked to hyper- and hypoglycemia risk and metabolic instability [19]. We quantify GV using the coefficient of variation (CV), defined as the standard deviation of glucose values divided by the mean glucose value and expressed as a percentage. All three metrics were computed at the day level for each participant and then averaged across all days on record to obtain participant-level summaries.

**Table 1:** Overview of three publicly available datasets used for reproducibility analysis. Our final study subset included 128 participants with over 1.36 million glucose samples^1^.

	Attribute	OhioT1DM	DiaTrend	T1DEXI
**Full Data**	# Participants	12	54	497
**Study Subset**	# Participants	12	53	63
	Gender (M/F)	7/5	17/36	18/45
	**Age (years)**			
	Age (range)	20–80	19–74	21–67
	Mean Age (SD)	NR	32 (15)	37 (13)
	**Hemoglobin A1C (%)**			
	HbA1C range	NR	6.3–10	4.8–8.3
	Mean HbA1C (SD)	NR	7.8 (0.8)	6.6 (0.8)
	**Insulin Delivery Mode**			
	Multiple Daily Injection	–	–	36.5%
	Insulin Pump / Closed Loop System	100%	100%	63.5%
	**Blood Glucose Data**			
	CGM Days	652	2,654	1,755
	Total BG Samples	166,533	709,555	487,287
	Average daily CGM coverage (%)	88.7%	92.8%	96.4%
	Mean (SD) of daily avg. BG per person (mg/dL)	159.0 (33.0)	182.4 (31.6)	145.7 (32.3)
	Mean (SD) of daily TIR per person (%)	63.0 (21.2)	54.5 (16.2)	73.4 (19.8)
	Mean (SD) of daily GV per person	31.2 (9.1)	31.1 (4.8)	30.1 (8.8)
	**Other lifestyle factors**			
	Total meal logs	2,168	9,096	10,376
	Total bolus records	3,733	21,273	11,067
	Total days with steps^2^	322	–	2,036
	Mean (SD) of daily meal logs per person	3.34	3.33	6.07
	Mean (SD) of daily bolus record per person	5.85	7.79	6.36
	Mean (SD) of daily steps per person	887	–	7,491

Notes. ^1^NR = not reported. Dashes (**–**) indicate not applicable or not available. TIR means time in range. GV means glycemic variability.

^2^Only six participants in OhioT1DM from the 2018 release have steps data in record.

**OhioT1DM** is the most widely used benchmark in prior studies [20–27], however, it consists of a small but intensively monitored cohort (n = 12). This dataset contains multimodal data collected from wearable devices and manual user logs, including CGM measurements recorded at 5-minute intervals, basal and bolus insulin delivery records from insulin pumps, self-reported meal times, meal types, and estimated carbohydrate content, step count data from the Basis Peak sensor band, as well as additional physiological and behavioral information, such as sleep quality, stress levels, and heart rate. With respect to glycemic control, participants in this dataset exhibit moderate glucose management, with an average daily TIR of 63% (clinical target: TIR > 70% [30]).

**DiaTrend** comprises data from 54 individuals with type 1 diabetes (T1D) who demonstrate more heterogeneous and suboptimal glucose control, with an average daily TIR of 55% (clinical target: TIR > 70% [30]). The DiaTrend dataset includes data from CGMs and insulin pumps, comprising CGM measurements sampled at 5-minute intervals. However, basal and bolus insulin delivery records are only available for the final 1–2 months of the data collection period.

**Fig 2:**
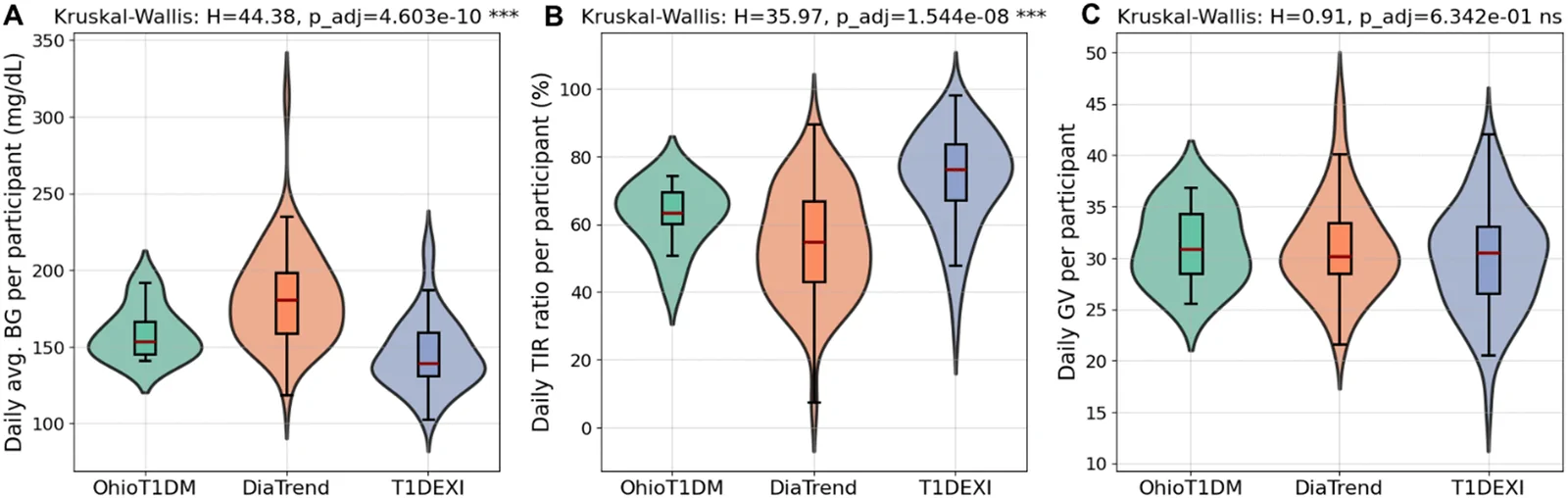
Distribution of key metrics for assessing blood glucose management in three publicly available datasets - OhioT1DM [12], DiaTrend [14], and T1DEXI [15]. **A)** Daily average blood glucose per participant, **B)** Daily time in range ratio per participant, and **C)** Daily glycemic variability per participant.

In contrast, **T1DEXI** features a larger, better-managed, and more physically active cohort, with an average daily TIR of 73%, (clinical target: TIR > 70% [30]). This dataset includes 5-minute interval CGM time-series data, step count, and physical activity information recorded by the Verily Study Watch, basal and bolus insulin delivery records from insulin pumps or multiple daily injections (MDI), and self-reported meal intake information. Fig 2 shows that these datasets collectively span a wide spectrum of glycemic control and further highlight participant-level differences across average daily BG, daily TIR, and daily glycemic variability. Therefore, including these three datasets offers an opportunity to examine model reproducibility and generalization across varying diabetes management conditions.

Although these three datasets contain multiple data modalities from different sources, this study mainly used the CGM time-series data. The CGM devices differed across datasets: the OhioT1DM dataset was collected with Medtronic Enlite CGM sensors, DiaTrend was collected with CGMs from Dexcom, Abbott, and Medtronic, while T1DEXI primarily included Dexcom G6 CGMs. All CGMs here comply with ISO 15197:2013 and FDA accuracy standards, requiring ≥95% of measurements to fall within ±15 mg/dL (for glucose <100 mg/dL) or ±15% (for glucose ≥100 mg/dL) [26]. These systems are widely used clinical standard for managing type 1 diabetes; therefore, the impact of device-specific variability is expected to be minimal.

To account for differences in dataset structure, annotation availability, and recording duration across the three datasets, we applied dataset-specific inclusion and harmonization criteria. The goal was to ensure that each included participant had sufficient CGM continuity and relevant event annotations for the intended analyses, while also preventing any single dataset or participant from disproportionately influencing the results. For **OhioT1DM**, we retained the original 12 participants and preserved the official train–test split provided by the dataset creators. For **DiaTrend**, we included 53 of the 54 available participants, excluding one participant (#52) with fewer than 28 days of contiguous CGM data, and restricted each participant to the most recent eight weeks of data to reduce imbalance caused by participants with years of recordings and to prioritize periods with more consistent meal and bolus annotations. For **T1DEXI**, we selected participants with good data consistency–at least seven consecutive days of ≥70% CGM coverage and at least one meal and bolus record per day; this yielded a subset of 63 participants, providing sufficient event-annotated data while maintaining a sample size comparable to DiaTrend and OhioT1DM. Across all datasets, CGM data were harmonized to a 5-minute sampling interval when needed. The selected participants’ IDs were listed in S1 Table.

### Model selection and characteristics

In this study, we sought to investigate the technical, statistical, and conceptual reproducibility of several SOTA deep learning methods for blood glucose prediction. To select high-quality methods from prior work for reproducibility evaluation, we considered the following four criteria: (1) code availability and/or detailed model reporting, (2) evaluation on the publicly available OhioT1DM dataset, (3) strong performance for short-term prediction (30 min), and (4) diversity of model architectures. More specifically, we prioritized studies that provided either publicly accessible code or sufficiently detailed methodological descriptions to support faithful reimplementation. We also restricted model selection to studies that evaluated performance on the OhioT1DM dataset to enable direct comparison between the originally reported results and our reproduced results. As the most widely used publicly available benchmark in glucose forecasting research, OhioT1DM serves as a common reference point for methodological comparison (Fig 1), providing a consistent basis for assessing technical, statistical, and conceptual reproducibility. To ensure consideration of high-quality methods for short-term glucose forecasting, we also required strong reported performance at the 30-minute prediction horizon on OhioT1DM (i.e., RMSE < 20 mg/dL). Finally, to capture the dominant modeling paradigms in prior literature, we selected model architectures that are LSTM-based, CNN-based, and Transformer-based, which account for 43%, 13%, and 12%, respectively, of model architectures used in prior work based on our literature review (see S1 File).

Leveraging the above criteria, we selected and experimentally replicated eight representative studies: three LSTM-based models—Martinsson et al. (2019) [20], van Doorn et al. (2021) [21], and Rabby et al. (2021) [24]; two CNN-based models—Li et al. (2020) [22] and Deng et al. (2021) [23]; and three Transformer-based models—Lee et al. (2023) [25], Zhu et al. (2025) [26], and the PatchTST implementation in Karagoz et al. (2025) [27]. Table 2 summarizes the eight selected studies in terms of their datasets used for training and evaluation, input features, code availability, and model type. Although these models share certain methodological and implementation similarities, they also exhibit notable differences across datasets, input configurations, and evaluation criteria. With respect to datasets, all eight studies evaluated their proposed models on the OhioT1DM dataset. However, there were some differences in their evaluation strategy. For example, some studies trained and tested their models on only a subset of the dataset, specifically the first six participants released in 2018 [20–22, 24], while others used the full set of twelve participants [23]. In contrast, some other studies trained their models on external datasets and used OhioT1DM solely for evaluation [25–27]. Furthermore, some studies incorporated additional datasets during model training. For example, Li et al. [22] used the ABC4D dataset to incorporate more participants with type 1 diabetes (T1D), while van Doorn et al. [21], Deng et al. [21], and Lee et al. [25] included individuals with type 2 diabetes (T2D) during pretraining to enhance data diversity. However, when evaluating on the OhioT1DM dataset, these studies applied transfer learning or retraining procedures to adapt the models specifically to T1D data. Therefore, despite differences in pretraining data composition, all selected models were ultimately evaluated under a T1D setting and remained appropriate for our reproducibility analysis.

Regarding input features, five studies relied exclusively on historical CGM data [20,21,23,25,26], whereas others incorporated additional contextual data such as insulin delivery, carbohydrate intake, or step count [22,24,27] as shown in Table 2. Differences also emerged in evaluation metrics. While most studies reported performance across multiple metrics and prediction horizons, the most common evaluation metric used was the Root Mean Squared Error (RMSE) between predicted and actual glucose values at a 30-minute prediction horizon - see Eq 1. Exceptions include Lee et al. (2023) [25], which reported only mean absolute percentage error (MAPE), and Karagoz et al. (2025) [27], which computed RMSE over the entire prediction sequence rather than at the fixed 30-minute prediction horizon, as shown in Eq 2. This sequence-level RMSE can yield lower reported errors by averaging performance across earlier and typically easier prediction steps. By contrast, *RMSE*_30_ reflects the error at the clinically relevant 30-minute prediction horizon and is consistent with best-practice guidelines and recent literature reviews [11, 31]. Therefore, in our reproducibility evaluation, we selected the sampling horizon (i.e., the duration of historical input data) with the best reported performance in each prior study for replication, and used *RMSE*_30_ as the primary evaluation metric. More detailed model specifications and implementation information are provided in S2 Table and S2 File.


$$\begin{array}{c} {\text{RMSE}}_{30}=\sqrt{\frac{1}{N}\sum_{i=1}^{N}({\hat{y}}_{i,t+30}-y_{i,t+30}{)}^{2}} \end{array}$$ (1)

$$\begin{array}{c} {\text{RMSE}}_{\text{seq}}=\sqrt{\frac{1}{N}\sum_{i=1}^{N}\frac{1}{L}\sum_{j=1}^{L}({\hat{y}}_{i,t+j}-y_{i,t+j}{)}^{2}} \end{array}$$ (2)

where *N* is the number of test samples and *L* is the prediction sequence length (*L* = 30 minutes in this study).

**Table 2:** Summary of the eight deep learning studies selected for replication. The table lists each study’s input features, training and evaluation datasets, code availability, sampling horizons, model architecture, and major reported metrics.

Method	Input Features^1^	Training Dataset^2^	Evaluation Dataset	Open Code	Sampling Horizon	Model Architecture	Reported Metrics
Martinsson 2019 [20]	G	Ohio18 Train	Ohio18 Test	✓	60 min	LSTM	*RMSE*_30_
Li 2020 [22]	G, C, I	Ohio18 Train	Ohio18 Test	×	80 min	Dilated CNN	*RMSE*_30_
van Doorn 2021 [21]	G	Ohio18 Train	Ohio18 Test	×	30 min	Dual LSTM	*RMSE*_30_
Deng 2021 [23]	G	Ohio Train	Ohio20 Test	✓	30 min	Gated CNN + TL^3^	*RMSE*_30_
Rabby 2021 [24]	G, C, I, S	Ohio18 Train	Ohio18 Test	×	120 min	Stacked LSTM	*RMSE*_30_
Lee 2023 [25]	G	Private	Ohio Train&Test	×	60 min	TransformerEncoder	MAPE
Zhu 2025 [26]	G	REPLACE-BG [28]	Ohio Test	Part	6 hrs	Transformer	*RMSE*_30_
Karagoz 2025 [27]	G, C, I	DCLP3 [29]	Ohio Test	Part	4 hrs	PatchTST [30]	*RMSE*_seq_

Notes. ^1^G: glucose; C: carbohydrate; I: insulin; S: step count.

^2^Ohio18: Six participants in the OhioT1DM dataset released in 2018. Ohio20: Six participants from the OhioT1DM dataset released in 2020. Ohio: The full OhioT1DM dataset, combining both the 2018 and 2020 releases.

^3^TL: Transfer learning.

In addition to the differences summarized in Table 2, differences in the input feature set, model architecture, and input horizon also influenced how missing data was handled. For LSTM- and CNN-based models or models using shorter input horizons with CGM-only inputs [20,21,23,25], predictions were limited to available continuous CGM segments to preserve temporal consistency. In contrast, models that incorporated additional lifestyle features (e.g., insulin, carbohydrate, or activity) [22,24], as well as Transformer-based models with longer input horizons [26,27], addressed missing data through linear or spline interpolation within sequences and extrapolation for terminal gaps to maintain sequence continuity. Therefore, when replicating these works, the input data were interpolated as the original studies did.

## Reproducibility evaluation framework

### Technical reproducibility evaluation

Technical reproducibility refers to the ability of a model’s result to be fully replicated under identical experimental conditions [13]. To evaluate the technical reproducibility of the eight deep learning models, we sought to reproduce them using the same experimental settings, including the same training and evaluation dataset, exact input features, missing data handling process, model architecture, training, validation, and test logic, and evaluation metric reported in the original publication. In other words, regardless of the methodological differences summarized in Table 2, we reproduced each study’s reported results by following either the authors’ released code or the implementation details described in the paper. However, the inconsistencies existing throughout the entire pipeline make direct comparison between studies difficult, highlighting the necessity for more standardized and aligned evaluation protocols.

### Statistical reproducibility evaluation

Statistical reproducibility refers to the ability of a model’s result to be “upheld under resampled conditions that may yield mildly different numerical results but should not statistically affect the claimed result” [13]. In this study, rather than resampling, which would further reduce the already limited OhioT1DM dataset, we trained all eight models using the complete training portion of the OhioT1DM dataset and evaluated them on its test portion. For some studies [20–24], this adjustment effectively acted as an “upsampling,” incorporating a broader range of training instances from the same dataset. However, for studies originally trained on external datasets [25–27], the change primarily served to maintain consistency between training and evaluation setups by preserving a comparable data distribution. This unified experimental setting ensures that observed performance differences reflect variations in model design and implementation rather than inconsistencies in data usage. While this approach differs from the resampling-based definition, it serves as a practical adaptation of statistical reproducibility for a small, real-world biomedical dataset. In addition, we refined the input configurations of models that originally incorporated multiple input factors [22,24,27] by comparing their technical replication results using either a single CGM input channel or multiple channels integrating additional lifestyle features, as shown in S3 Table. For the statistical reproducibility evaluation, the implementation of Karagoz et al. (2025) remained multi-channel, whereas Li et al. (2020) and Rabby et al. (2021) were optimized to use a single CGM input channel due to better performance during the technical reproducibility evaluation.

In addition to unifying and optimizing the model inputs, the statistical reproducibility evaluation also standardized the output metrics to the conventional RMSE at a fixed 30-minute prediction horizon, as defined in Eq 1. Overall, the implementation for statistical reproducibility evaluation unifies the input and evaluation metrics for the eight methods, while maintaining the consistency and integrality of the blood glucose prediction pipeline. This standardization further ensured comparability across methods and allowed observed performance differences to be attributed primarily to variations in model architecture and learning dynamics.

### Conceptual reproducibility evaluation

Conceptual reproducibility describes whether the intended effect of a model can be reproduced under conditions that align with its high-level purpose [13]. To assess conceptual reproducibility, we evaluated the same eight methods on two additional publicly available datasets—DiaTrend [14] and T1DEXI [15]. Both datasets include individuals with type 1 diabetes but differ substantially from OhioT1DM in cohort size and overall glycemic control (Table 1, Fig 2). All models were independently retrained on the DiaTrend and T1DEXI datasets, respectively, to ensure dataset-specific learning rather than transfer from OhioT1DM. For the DiaTrend and T1DEXI datasets, we performed 5-fold cross-validation, training on four folds and evaluating on the remaining fold, iterating until all participants were tested as unseen subjects. This setup ensured a fair comparison across models and directly assessed each model’s ability to generalize to new individuals. Apart from replacing the training and evaluation datasets (OhioT1DM) with DiaTrend and T1DEXI, and adopting a 5-fold cross-validation scheme, all other experimental settings were identical to those used in the statistical reproducibility evaluation described in the previous subsection.

## Results

This section presents a comprehensive evaluation of the reproducibility of eight deep learning models for blood glucose prediction following the aforementioned evaluation framework, including the results of technical, statistical, and conceptual reproducibility evaluation. We further analyzed how individual diabetes management factors and demographic factors contribute to variability in model performance across datasets, identifying the fundamental reason that strongly affects prediction accuracy.

### Technical reproducibility

The technical reproducibility evaluation examined whether the reported performance of each deep learning model for blood glucose prediction could be replicated under the same experimental settings as described in their original studies. As summarized in Table 3, the RMSE values reported across the seven studies (excluding Lee et al., 2023) ranged from 15.96 to 19.28 mg/dL, while our exact replication results ranged from 15.45 to 19.41 mg/dL. The results of Martinsson (2019), Deng (2021), Zhu (2025), and Karagoz (2025) were closely reproduced with minimal deviation likely attributable to inherent training stochasticity, whereas Li (2020), van Doorn (2021), and Rabby (2021) yielded slightly higher RMSEs but still within ±1SD of the reported values, indicating good overall reproducibility.

**Table 3:** Overview of technical and statistical reproducibility evaluation of eight deep learning papers on 30-minute blood glucose prediction. Paper reported RMSE (mg/dL) is the authors’ reported RMSE for future 30-min prediction with standard deviation (SD) in parentheses.

Method	Paper Reported RMSE (mg/dL)	Technical Reproducibility Evaluation Result	Statistical Reproducibility Evaluation Result
Martinsson 2019 [20]	18.87 (1.79)	18.76 (2.02)	18.86 (2.13)
Li 2020 [22]	19.28 (2.76)	20.34 (1.90)	19.31 (2.38)
van Doorn 2021 [21]	18.83 (NR)	20.22 (2.00)	19.20 (2.14)
Deng 2021 [23]	19.08 (NR)	19.41 (2.30)	19.34 (2.30)
Rabby 2021 [24]	18.57 (1.68)	20.35 (2.76)	19.78 (2.37)
Lee 2023 [25]	MAPE = 17.88% (NR)	–^4^	19.78 (2.45)^1^
Zhu 2025 [26]	18.9 (NR)	19.08 (3.10)	21.58 (2.63)
Karagoz 2025 [27]	15.96 (2.65)	15.45 (2.61)^2^	20.27 (3.43) ^3^

Notes. ^1^Corresponding MAPE ≈ 9.70%.

^2^The averaged RMSE and SD calculated with the conventional way *RMSE*_30*min*_ in Eq 1 is 22.06 (3.47)

^3^The RMSE and SD calculated by the custom way in Karagoz et al., 2025 $RMSE_{seq}$ in Eq 2 is 14.16 (2.54).

^4^Lee et al. trained the original model using a private dataset; therefore, the original training setting could not be exactly replicated, and technical reproducibility results are not reported.

### Statistical reproducibility

Table 3 presents our results from statistical reproducibility evaluation. We can observe minimal differences between the paper-reported RMSEs, technical and statistical reproducibility evaluation results, thereby showing stability across most models when retrained and evaluated under standardized conditions. Our statistical reproducibility evaluation facilitated a fair comparison of the eight blood glucose prediction pipelines by retraining and evaluating them on the standardized OhioT1DM dataset using unified evaluation metrics, as described in the Methods section. The five traditional LSTM- and CNN-based pipelines showed minimal performance variation (within ±1.21 mg/dL of their technical reproducibility results), suggesting strong robustness to unified data and metric conditions. Meanwhile, the Transformer-based model from Zhu et al. (2025) exhibited a slight performance degradation, as can be seen from the RMSE increase by 2.5 mg/dL under statistical reproducibility evaluation. However, we can observe that all methods are within 1 SD of the paper-reported RMSE and the technical reproducibility result. In contrast, from Table 3, we can observe that a discrepancy of 6.11 mg/dL between Karagoz et al. (2025)’s custom $RMSE_{seq}$ and the standardized *RMSE*_30*min*_, thereby underscoring how variations in metric definitions can substantially affect reported outcomes. These findings collectively suggest that while most models retain statistical reproducibility under controlled conditions, evaluation standardization is critical to enable fair comparisons across studies.

### Conceptual reproducibility

**Fig 3:**
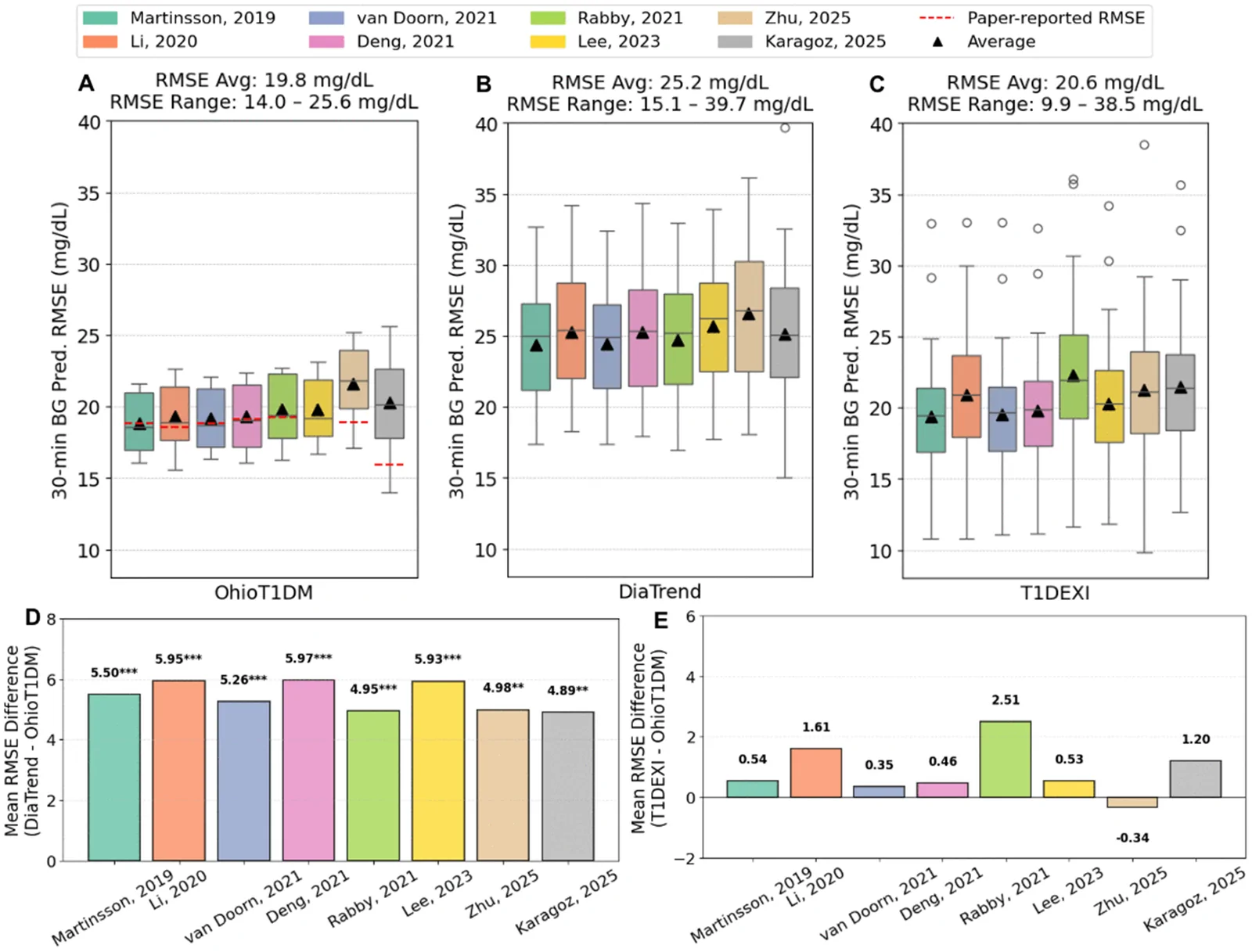
Reproducibility evaluation of eight deep learning methods from prior literature for the task of blood glucose prediction 30-minutes in advance. Evaluation results using three publicly available datasets, including **A)** OhioT1DM (n = 12), **B)** DiaTrend (subset: n = 53), **C)** T1DEXI (subset: n = 63). D & **E)** Present the differential performance of each blood glucose prediction method on the DiaTrend and T1DEXI datasets compared to the performance on the OhioT1DM dataset.

Fig 3 presents the results obtained from our evaluation of statistical (Fig 3A) and conceptual reproducibility (Fig 3B&C) for the eight deep learning papers evaluated in this study. From Fig 3B & C, we can observe varying levels of increased error in the prediction performance across all eight deep learning methods for blood glucose prediction when evaluated on DiaTrend and T1DEXI. More specifically, we observe differential performance with an increase in prediction error by 4.89 to 5.97 mg/dL on the DiaTrend dataset and -0.34 to 2.51 mg/dL on the T1DEXI dataset - see Fig 3D & E. Furthermore, we observe increased variability in prediction performance across all eight methods on DiaTrend and T1DEXI compared to OhioT1DM - see Fig 3A-C and S5 Table. In particular, the prediction performance across all eight methods ranged from 14.0 - 25.6 mg/dL RMSE per individual on OhioT1DM, compared to 15.1 - 39.7 mg/dL on DiaTrend, and 9.9 - 38.5 mg/dL on T1DEXI. We compared the difference in prediction performance for all eight methods on OhioT1DM vs. DiaTrend and OhioT1DM vs. T1DEXI using t-tests. Our results showed statistically significant differences in the prediction performance of all eight methods on the DiaTrend dataset compared to performance on the OhioT1DM dataset - see Fig 3D and S7 Table. These results indicate that while all deep learning models exhibited reasonable technical and statistical reproducibility, their conceptual reproducibility was limited. This limitation, however, may not arise solely from model design or prediction pipelines, but rather from intrinsic differences in glucose management behaviors across datasets—a factor further examined in the following section. Detailed RMSE and MAE values at 30-min prediction horizon can be found in S5 Table.

To examine cross-dataset robustness, we also evaluated OhioT1DM-pretrained models directly on DiaTrend and T1DEXI without retraining (S6 Table). We observed that most models exhibited modest performance degradation when using pretrained models on OhioT1DM, hence retraining each model provided the best performance.

### Prediction performance differs based on glucose management

As shown in Fig 3, replication RMSEs were consistently higher on the DiaTrend dataset than on OhioT1DM and T1DEXI. This pattern aligns with participants’ glycemic management profiles (Fig 2), where higher mean glucose and lower time-in-range are indicative of below target glycemic control. These observations suggest that prediction performance may be influenced by differences in individual glucose management.

**Fig 4:**
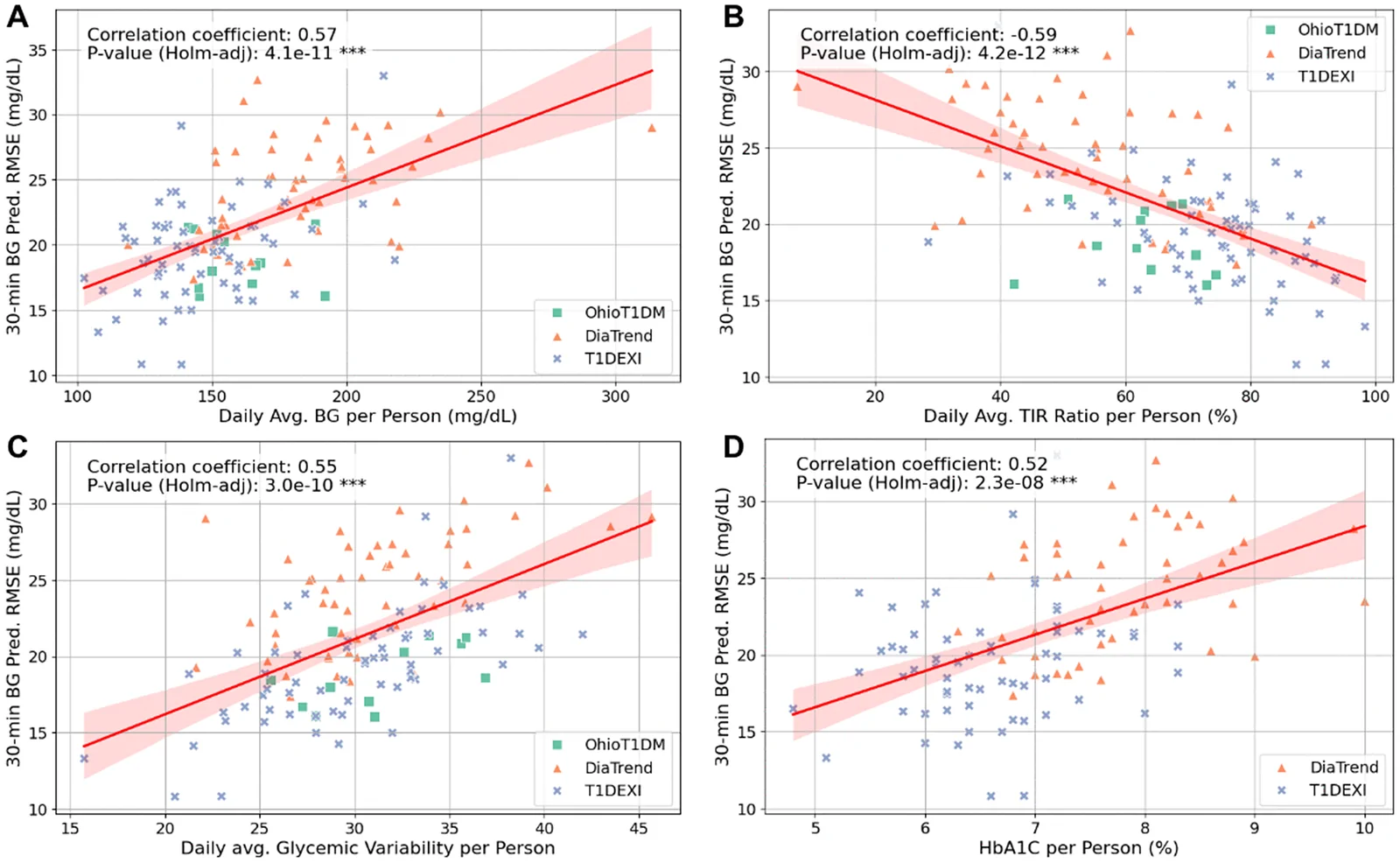
Association between blood glucose prediction and individual diabetes management. This figure uses the deep learning model proposed by Martinsson et al. [18] for illustration due to its best prediction performance across the eight models. **(A-D)** Blood glucose prediction RMSE vs. the daily average blood glucose per person, TIR per person, Glycemic variability per person, and HbA1C per person, respectively. Higher RMSE equates to greater error in the prediction performance.

To further evaluate potential associations between prediction performance and individual glycemic control, we quantified diabetes management of each person in all three datasets using four clinically-validated metrics, namely, mean glucose, time in range (TIR), glycemic variability, and HbA1C [17]. Each CGM metric (i.e., all metrics except HbA1C) was calculated on a daily basis, then an average across all days was calculated for each participant. Fig 4 illustrates the association between blood glucose prediction performance and individual-level diabetes management metrics. Across datasets and models, lower RMSE values (i.e., less prediction errors) were consistently observed in individuals with higher glycemic control (i.e., lower mean glucose, higher TIR, lower glycemic variability, and lower HbA1C). Additionally, higher prediction errors occurred amongst individuals with lower glycemic control (higher mean glucose, lower TIR, and higher glycemic variability). The Pearson correlation coefficients ranged from 0.38 to 0.63 (mean: 0.55; p < 0.001), indicating moderate-to-strong associations, with up to 40% of performance variance attributable to glycemic management.

These findings suggest that deep learning models perform substantially better in metabolically stable individuals characterized by smoother and more predictable glucose trajectories. In contrast, individuals with higher glycemic variability and lower TIR exhibit more abrupt fluctuations and nonlinear dynamics, which increase forecasting difficulty. Importantly, these findings suggest that current models may implicitly favor well-controlled patients, potentially overestimating robustness when evaluated with datasets from a stable cohort. The reduced accuracy observed in individuals with poorer glycemic control raises concerns, as these patients may benefit most from reliable glucose prediction. Overall, the results highlight that glycemic control is a critical determinant of predictive performance and should be explicitly considered in reproducibility and generalization assessments.

As supplementary assessments, we also examined whether demographic factors—including sex/gender, age, and insulin delivery mode—are associated with differences in blood glucose prediction performance across the eight deep learning methods. Specifically, we assessed the relationship between the listed demographic variables and prediction RMSE (S9 Table, S10 Table, and S11 Table) and conducted stratified analyses by diabetes management level to better isolate the sources of performance variability (S1 Fig). These analyses suggest that differences in glucose management, rather than demographic characteristics, account for most observed performance disparities. However, it is important to note our analysis is limited because there was a significant imbalance across sex/gender (33% male vs 67% female), age (48% < 30 years), and insulin delivery mode (82% insulin pump/closed loop). This imbalance limits the strength and generalizability of this finding. Therefore, until these patterns are validated in a larger and more demographically balanced cohort, we remain cautious about drawing strong conclusions regarding demographic effects.

## Discussion

This section presents a discussion on the challenges encountered during our reproducibility experiments and recommendations for future work.

### Challenges to reproducibility in blood glucose prediction

The experimental replication provides a concrete way to identify and understand the practical challenges that hinder reproducibility in blood glucose prediction research. Although we observed the consistency between the paper-reported results and the technical replication results from Table 3, several reproducibility challenges still can not be overlooked. These challenges can be broadly grouped into three categories: (1) data-related constraints, (2) implementation transparency challenges, and (3) inconsistent evaluation metrics. With regard to data-related constraints, many studies relied heavily on the OhioT1DM dataset, which includes only 12 participants, thereby limiting the generalizability of their results. Also, Lee et al. 2023 [25] used private datasets for model training, thereby making reproducibility evaluations by independent researchers infeasible. In addition to data-related limitations, incomplete or missing code further hampered our reproducibility evaluation studies. For example, four of the eight studies did not openly release their source code, and even when code was available, such as Zhu et al. 2025 [26] and Karagoz et al. 2025 [27], critical implementation details were missing, including data filtering procedures, preprocessing scripts, and specifications for input data construction. We also observed that for complex model architectures there were sometimes numerous unreported hyperparameters which further challenged our reproducibility studies. These missing pieces introduced challenges for replication and possibly introduced errors in the replicated result. Finally, inconsistency in evaluation introduced additional challenges when comparing the performance of results across SOTA methods. Some studies, such as Lee et al. 2023 [25] did not report standard metrics such as RMSE, while some studies, such as Karagoz et al. 2025 [27] customized their RMSE calculation, thereby making it difficult to compare performance across methods without experimental replication. These issues highlight the pressing need for greater transparency, standardized evaluation practices, and broader data accessibility to strengthen reproducibility and comparability in deep learning models for blood glucose prediction.

In addition to the findings from the technical reproducibility evaluation, the statistical reproducibility evaluation results shown in Table 3 reflect a fair comparison result between eight models, based on unified input and standardized evaluation metrics. As shown in Table 3, we observed that the three Transformer-based models achieved lower predictive performance compared with LSTM and CNN-based architectures. Similar findings have also been reported in some prior studies on time-series forecasting [32,33]. This discrepancy in prediction performance likely stems from multiple reasons. One possible reason is that those Transformer-based models rely on longer temporal windows, especially Zhu et al, 2025 [26] and Karagoz et al. 2025 [27], which have a higher requirement on data completeness and quality. Another possible reason is that the three replicated Transformer architectures have higher model complexity as shown in S2 Table, and usually require larger training datasets to achieve stable convergence [33]. Given the limited sample size of the OhioT1DM dataset (n = 12), these requirements may not be fully met, which may explain their suboptimal performance relative to the more data-efficient RNN- and CNN-based models.

Compared with the relatively stable results from the technical and statistical reproducibility evaluations, the conceptual reproducibility analysis revealed substantially lower generalizability when models were retrained on new datasets (Fig 3). The consistently higher RMSEs and variance observed on the DiaTrend and T1DEXI datasets across almost all eight methods suggest that differences in glycemic management strongly influence prediction performance. In particular, individual management heterogeneity (Fig 4) plays a decisive role: models tend to perform better for individuals with well-controlled glucose trends and worse for individuals with poorer glycemic control.

While the correlation analysis reported that demographic factors such as age and insulin delivery mode are highly relevant to prediction accuracy, a further stratified analysis (S1 Fig) did not find consistent evidence supporting strong demographic effects. Glucose management could play a more prominent role than demographics in shaping prediction performance, but further validation in larger and more balanced cohorts is needed to confirm this relationship. However, it is still worthwhile for future studies on blood glucose prediction to prioritize recruiting participants with diverse levels of glycemic control, not merely to improve model performance, but to enable fairer and more generalizable evaluations that better reflect real-world variability.

### Recommendations for future work

Given the replication challenges and the differential performance observed across each individual, across management subgroups, and across datasets, we present three core recommendations toward the goal of developing robust machine learning algorithms for blood glucose prediction.

- **Management-Aware Model Development** A central finding of this study is that individual glycemic management characteristics are highly correlated with models’ prediction performance. Future glucose prediction research should therefore explicitly incorporate management-aware modeling strategies. This may include conditioning models on metrics such as average glucose level, TIR, and/or glucose variability; designing adaptive architecture for participants with different management profiles; and routinely reporting performance stratified by glucose management levels. Such practices would help ensure that model performance is not overstated when evaluated on datasets from individuals with less stable glucose profiles and would support the development of models robust to the challenging patterns in the real world.
- **Dataset Transparency and Reporting Standards** Our results highlight the extent to which dataset characteristics can influence prediction performance. To ensure fair comparison across studies, we recommend adopting minimum dataset reporting standards tailored to glucose prediction tasks. These include reporting glycemic management metrics (e.g., mean glucose, TIR/TAR/TBR, glucose variability), CGM quality indicators (e.g., percentage of missing data, interpolation ratios, device type), and relevant behavioral context (e.g., meal regularity or insulin delivery mode when available). Standardized dataset characterization would not only improve transparency but also contextualize performance differences and aid reproducibility studies across research groups.
- **Data, Code, and Evaluation Transparency** To support fair comparison and reproducibility, future glucose prediction studies should adopt transparent and standardized practices in dataset selection, code release, and model evaluation. Whenever possible, models should be evaluated on publicly available datasets, with clear reporting of glycemic management characteristics to contextualize performance. Complete and well-organized code releases, including detailing model architectures, hyperparameters, and implementation choices, as these are essential for technical reproducibility evaluation. Studies should also report standardized metrics (e.g., RMSE) at benchmark prediction horizons such as 30 minutes ahead to enable meaningful cross-study comparison. Although these practices have become increasingly common in the SOTA, reiterating them is still worthwhile, as they remain foundational for transparent and rigorous methodological evaluation.

## Conclusion

This study set out to understand not only how well existing deep learning methods work for blood glucose prediction, but also how reliably these models are when conditions change. Toward this goal, we conducted reproducibility studies with eight representative deep learning methods and evaluated them across three public datasets. We followed a standardized reproducibility framework that traces a model’s journey from exact replication to broader generalization. Along this path, we found that while the paper-reported results of these models could be reproduced under consistent data and configurations, the performance of several deep learning models shifted once retrained on datasets with different diabetes management characteristics. We observed that prediction performance did not hinge primarily on pipeline/model architecture; instead, it was strongly influenced by how stable or unstable the glucose trajectories were for different individuals. Individuals with more glycemic fluctuations or suboptimal glucose control consistently posed greater challenges to all models. This study also highlights broader reproducibility challenges within the field, including limited datasets and accessibility, incomplete reporting of modeling choices, and inconsistent evaluation metrics. These gaps make it difficult to trace why models succeed in some settings and fail in others. In conclusion, strengthening reproducibility practices through transparent documentation, standardized evaluation pipelines, and more diverse data resources will be essential for moving glucose prediction research toward models that are not only accurate but also dependable across the varied realities of diabetes management.

### Code Availability and Environment

The source code used for this study is publicly available at https://github.com/Augmented-Health-Lab/ReproducibilityStudy_DL_BGPrediction. Detailed instructions for running the experiments and replicating the analysis are provided in the associated README file.

All code developed and used in this study is based on Python 3.9.20. For all machine learning tasks, PyTorch 2.2.1 + CU118, TensorFlow 2.10.1, and scikit-learn 1.5.2 were used. Data analysis and statistics were conducted with SciPy 1.11.4, NumPy 1.26.2 and Pandas 2.1.3.

## Supporting information

S1 File Literature review summary and study selection details. (XLSX)

S2 File Implementation details of the selected studies. (PDF)

S1 Table List of de-identified subject IDs included from the T1DEXI dataset. (PDF)

S2 Table Model and training details described in the original works of the eight selected deep learning methods for blood glucose prediction. (PDF)

S3 Table Comparing blood glucose prediction models trained with CGM plus other lifestyle factors (i.e., carbohydrate input, insulin data, and step count) versus CGM only as input features. These methods were trained and evaluated using the OhioT1DM dataset.
(PDF)

S4 Table Overall RMSE (mg/dL) for predicting blood glucose 30 mins ahead using a zero-order hold baseline. (PDF)

S5 Table Eight model RMSE and MAE in future 30-min prediction horizon on three datasets: OhioT1DM, DiaTrend, and T1DEXI. (PDF)

S6 Table External validation of eight OhioT1DM-pretrained models on DiaTrend and T1DEXI (30-minute RMSE). (PDF)

S7 TableWelch’s *t*-test comparing per-subject retrained-model errors across datasets. *diff* values are the RMSE difference between OhioT1DM and the other dataset ($RMSE_{DiaTrend}-RMSE_{OhioT1DM}$ or $RMSE_{T1DEXI}-RMSE_{OhioT1DM}$). *p*_adj_ values are Holm–Bonferroni corrected across the family of 16 tests (8 methods × 2 dataset pairs).Significance: *** *p*_adj_ < 0.001, ** *p*_adj_ < 0.01, * *p*_adj_ < 0.05.(PDF)

S8 TableCorrelation between individual diabetes management indicators (Avg.BG, TIR, GV, and HbA1c) and RMSE (mg/dL) for predicting blood glucose 30 minutes ahead. Pearson *r* pooled across all subjects from OhioT1DM, DiaTrend, and T1DEXI (*n* = 128; HbA1c excludes OhioT1DM, *n* = 113). *p*-values are Holm–Bonferroni corrected across the family of 32 tests (8 methods × 4 indicators); all are highly significant (*p* < 0.001).(PDF)

S9 Table Difference in 30-min prediction RMSE between male (*n* = 42) and female (*n* = 86) subgroups, pooled across OhioT1DM, DiaTrend, and T1DEXI. Welch’s two-sample *t*-test with *p*_adj_ Holm–Bonferroni corrected across the family of 8 method tests. Significance: *** *p*_adj_ < 0.001, ** *p*_adj_ < 0.01, * *p*_adj_ < 0.05.
(PDF)

S10 Table Comparison of 30-min prediction RMSE (mg/dL) across age groups and corresponding one-way ANOVA results. Subjects pooled across OhioT1DM, DiaTrend, and T1DEXI (*n* = 128); categorical age ranges binned by midpoint. *p*_adj_ values are Holm–Bonferroni corrected across the family of 8 method tests. Significance: *** *p*_adj_ < 0.001, ** *p*_adj_ < 0.01, * *p*_adj_ < 0.05.
(PDF)

S11 Table Comparison of 30-min prediction RMSE (mg/dL) across insulin delivery modes and corresponding one-way ANOVA results. Subjects pooled across OhioT1DM, DiaTrend, and T1DEXI (*n* = 128). *p*_adj_ values are Holm–Bonferroni corrected across the family of 8 method tests. Significance: *** *p*_adj_ < 0.001, ** *p*_adj_ < 0.01, * *p*_adj_ < 0.05.
(PDF)

S1 FigComparison of 30-minute blood glucose prediction RMSE across different demographic factors within glycemic management groups.Glucose management groups were defined based on each participant’s averaged daily time-in-range (TIR) ratio. Participants with TIR ratio <50% were assigned to the poor management group, those with 50%≤ TIR ratio <70% were assigned to the moderate management group, and those with TIR ratio ≥70% were assigned to the good management group. Within each management group, we compared 30-minute prediction RMSE by sex using independent two-sample *t*-tests. We also compared RMSE across age categories and insulin delivery modes using one-way ANOVA. For each family of subgroup tests, raw *p*-values were adjusted using the Holm–Bonferroni correction to account for multiple comparisons. (A) Gender-based differences (male vs. female). (B) Age-based differences across four age cohorts (<30, 30–40, 40–60, and 60–80 years). (C) Differences across insulin delivery modes, including multiple daily injections (MDI), pump, and closed-loop (CL) systems.(TIF)
