# Supplementary material for: Deep learning for blood glucose prediction: Reproducibility challenges and factors affecting differential performance
Source: PLOS Digit Health. 2026 Sep 3;5(9):e0001633. doi: 10.1371/journal.pdig.0001633 (PMC13541153; doi:10.1371/journal.pdig.0001633)
Supplement: Supplementary file 1 [file pdig.0001633.s002.pdf]

Implementation details of the selected studies

Martinsson et al., 2019 [17]

Martinsson et al. proposed a blood glucose prediction method that leverages a long short-term memory (LSTM) neural network. The proposed architecture includes one LSTM layer with 16 units (hidden size) followed by two fully connected layers with 512 and 256 units. The model was trained and evaluated using blood glucose data from the OhioT1DM dataset released in 2018 (n=6) and based on the dataset's original 80-20 training-test split. This model utilized a 60-minute history of CGM data to predict future blood glucose values. Martinsson et al. reported an average RMSE of 18.87 mg/dL for predicting blood glucose 30 minutes in advance.

Li et al., 2020 [19]

Li et al. proposed GluNet, a deep neural network (DNN) framework for short-term blood glucose prediction. The proposed architecture consists of a dilated CNN with five convolutional layers (32, 32, 64, 64, 128 filters) and a fully connected dense layer with 256 neurons. Dilation rates of 1, 2, 4, and 8 were used. The model was evaluated on 10 simulated patients with T1D from the UVA/Padova T1D simulator, 10 real-world participants with T1D from the ABC4D project, and 6 participants with T1D from the OhioT1DM dataset. The input features include 80 minutes of historical blood glucose data, meal intake, and both basal and bolus insulin data. Li et al. reported an average RMSE of 19.28 mg/dL for predicting blood glucose 30 minutes in advance.

van Doorn et al., 2021 [18]

van Doorn et al. proposed a deep learning model comprising two LSTM layers with 32 and 16 units, along with one dense layer containing 8 units. The model was primarily trained on the Maastricht Study dataset, and it was further evaluated on the OhioT1DM dataset released in 2018 (n=6). The model based on the OhioT1DM dataset utilized a 30-minute history of prior CGM data to predict future blood glucose values. The training-test split adhered to the original split in the OhioT1DM dataset. van Doorn et al. reported an average RMSE of 18.83 mg/dL for predicting blood glucose 30 minutes in advance on the OhioT1DM dataset.

Deng et al., 2021 [20]

Deng et al. introduced a deep learning approach that combines a gated convolutional neural network (CNN) with transfer learning for blood glucose prediction. Their proposed model architecture includes a neural network with 4 gated residual blocks, each containing 2 convolutional layers with 4 filters and a gating mechanism using sigmoid activation, followed by 2 fully connected layers with 10 units per layer. The model was trained on the OhioT1DM training dataset released in 2020 (n=12), but was only evaluated on the test dataset of the 6 participants released in 2020. The model uses a 30 minutes history of CGM data to predict future blood glucose values 30 minutes ahead. For each prediction target, they trained a population model using data from other participants in the dataset and then used transfer learning to fine-tune this model using the first 80% of CGM data in the training dataset from each target individual. The best performance reported by Deng et al. was an average RMSE of 19.08 mg/dL for predicting blood glucose 30-minute in advance on the OhioT1DM data subset.

Rabby et al., 2021 [21]

Rabby et al. developed a stacked LSTM-based recurrent neural network (RNN) model. Their proposed architecture consists of two LSTM layers with an intermediate dropout layer. The output from the second LSTM layer serves as input to a multi-layered fully connected network, which comprises two dense layers with 512 and 218 neurons, respectively, followed by a single-neuron output layer. The input data used for model training includes blood glucose data from a 120-minute historical window, self-reported meal logs, bolus insulin doses, and step count data. Additionally, the authors introduced a Kalman smoothing-based preprocessing technique to address sampling errors in the CGM data and denoise the raw data. However, their evaluation in the original study was conducted using smoothed blood glucose data, thereby significantly yielding a significantly lower RMSE of 6.45 mg/dL. In this study, our evaluation uses raw CGM data and eliminates the Kalman smoothing in an effort to ensure comparable assessment with other methods.

Lee et al., 2023 [22]

Lee et al. proposed a transformer-based model for blood glucose prediction. The model processes CGM data, using the past 60 minutes (12 steps) of blood glucose readings sampled every 5 minutes as input. These time-series inputs are first transformed into 512-dimensional vectors, to which sinusoidal positional encoding is added to incorporate temporal information. The core encoder layer consists of four parallel multi-head self-attention mechanisms and feed-forward sublayers, each followed by residual connections and layer normalization. The model outputs a single glucose value prediction 30 minutes ahead via a fully connected regression head. In the original study, the model was evaluated on the OhioT1DM dataset released in 2020 (n=12), and in accordance with the provided train-test split. Lee et al. reported an average MAPE of 17.88% for predicting blood glucose 30 minutes in advance on the OhioT1DM dataset. This study did not report RMSE.

Zhu et al., 2025 [23]

Zhu et al. proposed GPFormer, a Transformer-based population-level glucose prediction model designed for multi-horizon (future 30, 60, 90, 120 minutes) forecasting on type 1 diabetes. The model adopts a customized sparse multi-head self-attention (MHSA) mechanism within a Transformer encoder–decoder architecture and integrates a meta-learning domain generalization (MLDG) framework to improve robustness across datasets. In addition, a quantile loss function is incorporated to enhance the prediction of adverse glycemic events. The population model was trained on the large-scale REPLACE-BG dataset (n=226) using subject-level data partitioning and evaluated on three external datasets, including OhioT1DM. For 30-minute glucose prediction, GPFormer achieved an RMSE of 18.9 $\pm$ 3.2 mg/dL on the OhioT1DM dataset and consistently outperformed baseline models across multiple datasets and prediction horizons.

Karagoz et al., 2025 [24]

Karagoz et al. conducted a comparative evaluation of several transformer-based architectures for blood glucose prediction using multivariate inputs, including CGM, insulin, and meal information. Among the evaluated models, PatchTST [27]—a patch-wise Transformer architecture—demonstrated competitive short-term forecasting performance. PatchTST employs a channel-independent design, treating each variable

as a separate univariate series, and divides the input time series into overlapping patches that are processed through a shared Transformer backbone. This patch-based representation reduces computational complexity while enabling effective modeling of temporal dependencies. The models were trained on the DCLP3 dataset (n=112) and externally evaluated on the OhioT1DM dataset (n=12). For 30-minute glucose prediction on OhioT1DM, PatchTST achieved an RMSE of approximately 15.96 mg/dL, demonstrating strong short-term forecasting capability among the compared transformer variants.
