# Supplementary material for: Deep learning for blood glucose prediction: Reproducibility challenges and factors affecting differential performance
Source: PLOS Digit Health. 2026 Sep 3;5(9):e0001633. doi: 10.1371/journal.pdig.0001633 (PMC13541153; doi:10.1371/journal.pdig.0001633)
Supplement: Supplementary file 2 [file pdig.0001633.s003.pdf]

S1 Table . List of de-identified subject IDs included from the T1DEXI dataset.

1	11	18	24	25	103
114	115	144	152	173	187
248	252	1010	1015	1043	1082
1115	1121	1127	1139	1143	1171
1194	1201	1205	1211	1219	1230
1239	1271	1286	1311	1330	1336
1343	1345	1348	1361	1362	1363
1377	1381	1386	1408	1422	1427
1433	1435	1457	1459	1484	1503
1554	1558	1636	1650	1683	1689
1695	1722	1726			
