# Supplementary material for: Deep learning for blood glucose prediction: Reproducibility challenges and factors affecting differential performance
Source: PLOS Digit Health. 2026 Sep 3;5(9):e0001633. doi: 10.1371/journal.pdig.0001633 (PMC13541153; doi:10.1371/journal.pdig.0001633)
Supplement: Supplementary file 3 [file pdig.0001633.s004.pdf]

S2 Table . Model and training details described in the original works of the eight selected deep learning methods for blood glucose prediction.

Model Structure and Training Policy

Method	Model Type	Scaling	Train test split	Activation
Martinsson 2019 [15]	LSTM	0.01	80-20	exp
Li 2020 [17]	Dilated CNN	0.01	80-20	relu
van Doorn 2021 [16]	LSTM	0.01	80-20	relu
Deng 2021 [18]	CNN	0.01	80-20 + fine tune	relu
Rabby 2021 [19]	LSTM	0.01	80-20	relu + exp
Lee 2023 [20]	Transformer Encoder	LayerNorm	80-20	relu
Zhu 2025 [21]	Transformer	Standardization	REPLACE-BG(70/15/15)-OhioT1DM	GELU
Karagoz 2025 [22]	Transformer (PatchTST)	Instance-wise norm	DCLP3(80/10/10)-OhioT1DM	GELU

Network Architecture

Method	Network Layers
Martinsson 2019 [15]	1 LSTM (256) + 2 Dense (512, 256)
Li 2020 [17]	5 Conv (32, 32, 64, 64, 128)
van Doorn 2021 [16]	2 LSTM (32, 16) + 1 Dense (8)
Deng 2021 [18]	4 blocks with 2×1DConv + 2 Dense (10, 10)
Rabby 2021 [19]	2 LSTM (512 + 128 + 1)
Lee 2023 [20]	4 Multi-head attention encoders + Linear(512)
Zhu 2025 [21]	3 Encoders + 1 Decoder
Karagoz 2025 [22]	2 Encoders and 4 Attention heads($d_{model} = 64, d_{ff} = 128$)

Training Configuration

Method	Optimization	Loss Function	Regularization	Epochs
Martinsson 2019 [15]	Adam	NLL	DropOut(0.2, 0.3)	10000 (200)
Li 2020 [17]	Adam	RMSE	Early Stopping	100
van Doorn 2021 [16]	Adam	MSE	DropOut(0.1)	10000 (100)
Deng 2021 [18]	Adam	RMSE	L2 regularization	80
Rabby 2021 [19]	Adam	MSE	DropOut(0.2)	6000 (128)
Lee 2023 [20]	Adam	RMSE	Dropout(0.2)	200 (25)
Zhu 2025 [21]	SGD	Quantile Loss	weight decay=5×10^{-4}	50
Karagoz 2025 [22]	Adam	MSE	DropOut (0.1)	10

Method	Learning Rate	Batch Size
Martinsson 2019 [15]	10^{-3}	1024
Li 2020 [17]	10^{-4}	32
van Doorn 2021 [16]	10^{-4}	1024
Deng 2021 [18]	10^{-3}	64
Rabby 2021 [19]	10^{-3}	128
Lee 2023 [20]	10^{-3}, weight_decay=10^{-4}	64
Zhu 2025 [21]	10^{-3}	256
Karagoz 2025 [22]	10^{-3}	32
