# Supplementary material for: Deep learning for blood glucose prediction: Reproducibility challenges and factors affecting differential performance
Source: PLOS Digit Health. 2026 Sep 3;5(9):e0001633. doi: 10.1371/journal.pdig.0001633 (PMC13541153; doi:10.1371/journal.pdig.0001633)
Supplement: Supplementary file 4 [file pdig.0001633.s005.pdf]

S3 Table . Comparing blood glucose prediction models trained with CGM plus other lifestyle factors (i.e. carbohydrate input, insulin data, and step count) versus CGM only as input features. These methods were trained and evaluated using the OhioT1DM dataset.

	Mean RMSE at future 30min(mg/dL)	
Method	Using CGM + Others	Using CGM only
Rabby 2021 [23]	20.35	19.81
Li 2020 [21]	20.34	18.93
Karagoz 2025 [26]	20.27	21.76
