# Supplementary material for: Deep learning for blood glucose prediction: Reproducibility challenges and factors affecting differential performance
Source: PLOS Digit Health. 2026 Sep 3;5(9):e0001633. doi: 10.1371/journal.pdig.0001633 (PMC13541153; doi:10.1371/journal.pdig.0001633)
Supplement: Supplementary file 5 [file pdig.0001633.s006.pdf]

S4 Table. Overall RMSE (mg/dL) for predicting blood glucose 30 mins ahead using a zero-order hold baseline.

Public T1D Datasets	Mean	Std Dev	Max	Min
OhioT1DM [12]	23.21	2.89	28.40	18.81
DiaTrend [14]	28.72	5.28	42.50	20.78
T1DEXI [15]	24.14	5.06	40.32	13.47
