# Supplementary material for: Deep learning for blood glucose prediction: Reproducibility challenges and factors affecting differential performance
Source: PLOS Digit Health. 2026 Sep 3;5(9):e0001633. doi: 10.1371/journal.pdig.0001633 (PMC13541153; doi:10.1371/journal.pdig.0001633)
Supplement: Supplementary file 6 [file pdig.0001633.s007.pdf]

S5 Table. Eight model RMSE and MAE in future 30-min prediction horizon on three datasets: OhioT1DM, DiaTrend, and T1DEXI

Method	Mean	Std Dev	Max	Min	Mean	Std Dev	Max	Min
**OhioT1DM**	**RMSE**				**MAE**			
Martinsson 2019 [20]	18.86	2.13	21.63	16.04	13.31	1.50	15.74	11.42
Li 2020 [22]	19.31	2.38	22.63	15.56	13.51	1.56	16.06	11.33
van Doorn 2021 [21]	19.20	2.14	22.12	16.36	13.91	1.47	16.47	12.32
Deng 2021 [23]	19.34	2.30	22.35	16.10	13.70	1.49	16.11	11.60
Rabby 2021 [24]	19.78	2.37	22.74	16.25	14.48	1.66	17.22	11.92
Lee 2023 [25]	19.78	2.45	23.15	16.67	14.11	1.63	17.14	12.11
Zhu 2025 [26]	21.58	2.63	25.24	17.11	15.53	1.86	18.11	12.37
Karagoz 2025 [27]	20.27	3.43	25.64	13.99	13.57	2.35	16.59	8.05
**Aggregate Stats**	**19.76**	**2.48**	**25.64**	**13.99**	**14.02**	**1.69**	**18.11**	**8.05**
**DiaTrend**								
Martinsson 2019 [20]	24.35	3.77	32.69	17.37	17.52	2.63	23.82	12.50
Li 2020 [22]	25.25	4.06	34.22	18.30	18.37	2.86	25.26	13.56
van Doorn 2021 [21]	24.46	3.72	32.44	17.40	17.78	2.65	23.79	12.84
Deng 2021 [23]	24.24	4.18	34.36	17.91	18.29	2.89	23.96	13.23
Rabby 2021 [24]	24.73	3.86	32.95	16.95	17.78	2.75	23.96	12.03
Lee 2023 [25]	25.71	3.98	33.94	17.72	18.56	2.79	24.85	12.69
Zhu 2025 [26]	26.56	4.63	36.16	18.06	18.91	3.26	26.29	13.22
Karagoz 2025 [27]	25.16	4.51	39.66	15.04	16.96	3.31	25.31	6.77
**Aggregate Stats**	**25.19**	**4.09**	**39.67**	**15.05**	**18.02**	**2.89**	**26.29**	**6.77**
**T1DEXI**								
Martinsson 2019 [20]	19.40	3.79	32.98	10.81	13.91	2.64	22.70	7.66
Li 2020 [22]	20.92	4.33	33.03	10.81	15.43	3.35	22.25	7.71
van Doorn 2021 [21]	19.55	3.71	33.01	11.06	14.12	2.59	22.98	7.93
Deng 2021 [23]	19.80	3.73	32.63	11.13	14.20	2.59	22.45	8.00
Rabby 2021 [24]	22.25	4.59	36.08	11.62	16.90	3.78	28.10	8.47
Lee 2023 [25]	20.31	3.96	34.20	11.84	14.62	2.76	24.02	8.47
Zhu 2025 [26]	21.24	4.72	38.49	9.86	15.06	3.24	26.48	7.18
Karagoz 2025 [27]	21.47	4.22	35.64	12.69	15.11	2.97	24.84	8.68
**Aggregate Stats**	**20.62**	**4.13**	**38.49**	**9.86**	**14.92**	**2.99**	**28.10**	**7.18**
