# Supplementary material for: Deep learning for blood glucose prediction: Reproducibility challenges and factors affecting differential performance
Source: PLOS Digit Health. 2026 Sep 3;5(9):e0001633. doi: 10.1371/journal.pdig.0001633 (PMC13541153; doi:10.1371/journal.pdig.0001633)
Supplement: Supplementary file 7 [file pdig.0001633.s008.pdf]

S6 Table. External validation of eight OhioT1DM-pretrained models on DiaTrend and T1DEXI (30-minute RMSE)

Method	Mean	Std Dev	Max	Min
**DiaTrend: RMSE**				
Martinsson 2019 [20]	24.60	4.39	35.95	18.15
Li 2020 [22]	27.10	5.30	40.19	19.19
van Doorn 2021 [21]	24.53	4.26	35.51	17.88
Deng 2021 [23]	24.72	4.34	35.93	18.05
Rabby 2021 [24]	24.88	4.75	37.02	17.91
Lee 2023 [25]	25.43	4.61	36.91	18.01
Zhu 2025 [26]	37.84	6.78	51.13	25.35
Karagoz 2025 [27]	25.39	4.56	39.71	15.14
**T1DEXI: RMSE**				
Martinsson 2019 [20]	19.99	3.81	32.87	11.36
Li 2020 [22]	21.59	4.41	38.20	12.00
van Doorn 2021 [21]	20.08	3.73	33.06	11.54
Deng 2021 [23]	20.14	3.90	33.32	11.18
Rabby 2021 [24]	22.08	3.96	36.79	13.10
Lee 2023 [25]	20.70	4.05	35.32	12.25
Zhu 2025 [26]	31.40	6.75	52.88	16.79
Karagoz 2025 [27]	21.62	4.33	35.63	12.75
