# Supplementary material for: Deep learning for blood glucose prediction: Reproducibility challenges and factors affecting differential performance
Source: PLOS Digit Health. 2026 Sep 3;5(9):e0001633. doi: 10.1371/journal.pdig.0001633 (PMC13541153; doi:10.1371/journal.pdig.0001633)
Supplement: Supplementary file 8 [file pdig.0001633.s009.pdf]

S7 Table. Welch's t-test comparing per-subject retrained-model errors across datasets. $diff$ values are the RMSE difference between OhioT1DM and the other dataset ($RMSE_{DiaTrend} - RMSE_{OhioT1DM}$ or $RMSE_{T1DEXI} - RMSE_{OhioT1DM}$). p_{adj} values are Holm–Bonferroni corrected across the family of 16 tests (8 methods × 2 dataset pairs). Significance: *** $p_{\mathrm{adj}} < 0.001$, ** $p_{\mathrm{adj}} < 0.01$, * $p_{\mathrm{adj}} < 0.05$.

**Method**	**OhioT1DM vs DiaTrend**	**OhioT1DM vs T1DEXI**
Martinsson 2019 [20]	$diff = 5.50, t = -6.83$, $p_{\mathrm{adj}} = 2.52 \times 10^{-6}$ ***	$diff = 0.54, t = -0.69$, $p_{\mathrm{adj}} = 1.000$
Li 2020 [22]	$diff = 5.95, t = -6.72$, $p_{\mathrm{adj}} = 3.99 \times 10^{-6}$ ***	$diff = 1.61, t = -1.83$, $p_{\mathrm{adj}} = 0.546$
van Doorn 2021 [21]	$diff = 5.26, t = -6.56$, $p_{\mathrm{adj}} = 4.95 \times 10^{-6}$ ***	$diff = 0.35, t = -0.45$, $p_{\mathrm{adj}} = 1.000$
Deng 2021 [23]	$diff = 5.97, t = -6.80$, $p_{\mathrm{adj}} = 2.43 \times 10^{-6}$ ***	$diff = 0.46, t = -0.57$, $p_{\mathrm{adj}} = 1.000$
Rabby 2021 [24]	$diff = 4.95, t = -5.73$, $p_{\mathrm{adj}} = 5.35 \times 10^{-5}$ ***	$diff = 2.51, t = -2.80$, $p_{\mathrm{adj}} = 0.071$
Lee 2023 [25]	$diff = 5.93, t = -6.63$, $p_{\mathrm{adj}} = 5.86 \times 10^{-6}$ ***	$diff = 0.53, t = -0.61$, $p_{\mathrm{adj}} = 1.000$
Zhu 2025 [26]	$diff = 4.98, t = -5.03$, $p_{\mathrm{adj}} = 2.36 \times 10^{-4}$ ***	$diff = -0.34, t = 0.35$, $p_{\mathrm{adj}} = 1.000$
Karagoz 2025 [27]	$diff = 4.89, t = -4.19$, $p_{\mathrm{adj}} = 3.86 \times 10^{-3}$ **	$diff = 1.20, t = -1.07$, $p_{\mathrm{adj}} = 1.000$
