# Supplementary material for: Deep learning for blood glucose prediction: Reproducibility challenges and factors affecting differential performance
Source: PLOS Digit Health. 2026 Sep 3;5(9):e0001633. doi: 10.1371/journal.pdig.0001633 (PMC13541153; doi:10.1371/journal.pdig.0001633)
Supplement: Supplementary file 9 [file pdig.0001633.s010.pdf]

S8 Table. Correlation between individual diabetes management indicators (Avg. BG, TIR, GV, and HbA1c) and RMSE (mg/dL) for predicting blood glucose 30 minutes ahead. Pearson r pooled across all subjects from OhioT1DM, DiaTrend, and T1DEXI ($n = 128$; HbA1c excludes OhioT1DM, $n = 113$). p-values are Holm–Bonferroni corrected across the family of 32 tests (8 methods × 4 indicators); all are highly significant ($p < 0.001$).

**Method**	**Avg. BG vs RMSE** (r, $p_{\mathbf{adj}}$)	**TIR vs RMSE** (r, $p_{\mathbf{adj}}$)	**GV vs RMSE** (r, $p_{\mathbf{adj}}$)	**HbA1c vs RMSE** (r, $p_{\mathbf{adj}}$)
Martinsson 2019 [20]	$(0.48,\ 1.0\times10^{-7})$	$(-0.51,\ 1.4\times10^{-8})$	$(0.46,\ 4.5\times10^{-7})$	$(0.52,\ 8.4\times10^{-8})$
Li 2020 [22]	$(0.45,\ 6.6\times10^{-7})$	$(-0.49,\ 6.1\times10^{-8})$	$(0.47,\ 3.2\times10^{-7})$	$(0.50,\ 2.6\times10^{-7})$
van Doorn 2021 [21]	$(0.49,\ 9.3\times10^{-8})$	$(-0.52,\ 7.8\times10^{-9})$	$(0.47,\ 2.5\times10^{-7})$	$(0.52,\ 5.7\times10^{-8})$
Deng 2021 [23]	$(0.51,\ 2.1\times10^{-8})$	$(-0.53,\ 2.4\times10^{-9})$	$(0.45,\ 5.8\times10^{-7})$	$(0.55,\ 9.8\times10^{-9})$
Rabby 2021 [24]	$(0.40,\ 9.1\times10^{-6})$	$(-0.42,\ 2.7\times10^{-6})$	$(0.37,\ 2.8\times10^{-5})$	$(0.38,\ 3.3\times10^{-5})$
Lee 2023 [25]	$(0.52,\ 5.7\times10^{-9})$	$(-0.56,\ 2.2\times10^{-10})$	$(0.48,\ 1.8\times10^{-7})$	$(0.56,\ 2.9\times10^{-9})$
Zhu 2025 [26]	$(0.49,\ 9.3\times10^{-8})$	$(-0.54,\ 1.3\times10^{-9})$	$(0.53,\ 4.1\times10^{-9})$	$(0.52,\ 6.4\times10^{-8})$
Karagoz 2025 [27]	$(0.45,\ 6.1\times10^{-7})$	$(-0.49,\ 9.3\times10^{-8})$	$(0.52,\ 5.7\times10^{-9})$	$(0.45,\ 2.4\times10^{-6})$
