# Supplementary material for: Deep learning for blood glucose prediction: Reproducibility challenges and factors affecting differential performance
Source: PLOS Digit Health. 2026 Sep 3;5(9):e0001633. doi: 10.1371/journal.pdig.0001633 (PMC13541153; doi:10.1371/journal.pdig.0001633)
Supplement: Supplementary file 10 [file pdig.0001633.s011.pdf]

S9 Table. Difference in 30-min prediction RMSE between male ($n = 42$) and female ($n = 86$) subgroups, pooled across OhioT1DM, DiaTrend, and T1DEXI. Welch's two-sample t-test with p_{adj} Holm–Bonferroni corrected across the family of 8 method tests. Significance: *** $p_{\text{adj}} < 0.001$, ** $p_{\text{adj}} < 0.01$, * $p_{\text{adj}} < 0.05$.

	**30-min BG Pred. RMSE (mg/dL)**				
**Method**	**Male (n=42)** (Mean ± SD)	**Female (n=86)** (Mean ± SD)	**Difference**	***t*-statistic**	p_{adj}
Martinsson 2019 [20]	20.01 ± 4.13	22.08 ± 4.41	2.07	-2.60	0.066
Li 2020 [22]	21.27 ± 4.64	23.19 ± 4.57	1.92	-2.20	0.069
van Doorn 2021 [21]	20.23 ± 4.05	22.20 ± 4.35	1.97	-2.51	0.069
Deng 2021 [23]	20.64 ± 4.45	22.72 ± 4.69	2.08	-2.44	0.069
Rabby 2021 [24]	21.28 ± 4.30	23.93 ± 4.20	2.65	-3.30	0.012*
Lee 2023 [25]	20.98 ± 4.36	23.24 ± 4.70	2.26	-2.68	0.062
Zhu 2025 [26]	21.98 ± 4.93	24.21 ± 5.20	2.23	-2.36	0.069
Karagoz 2025 [27]	21.48 ± 4.97	23.58 ± 4.39	2.10	-2.33	0.069
