# Supplementary material for: Deep learning for blood glucose prediction: Reproducibility challenges and factors affecting differential performance
Source: PLOS Digit Health. 2026 Sep 3;5(9):e0001633. doi: 10.1371/journal.pdig.0001633 (PMC13541153; doi:10.1371/journal.pdig.0001633)
Supplement: Supplementary file 11 [file pdig.0001633.s012.pdf]

S10 Table. Comparison of 30-min prediction RMSE (mg/dL) across age groups and corresponding one-way ANOVA results. Subjects pooled across OhioT1DM, DiaTrend, and T1DEXI ($n = 128$); categorical age ranges binned by midpoint. p_{adj} values are Holm–Bonferroni corrected across the family of 8 method tests. Significance: *** $p_{\mathrm{adj}} < 0.001$, ** $p_{\mathrm{adj}} < 0.01$, * $p_{\mathrm{adj}} < 0.05$.

	**30-min BG Pred. RMSE (mg/dL)**					
**Method**	**<30yrs(n=62)**	**30–40yrs(n=22)**	**40–60yrs(n=35)**	**60–80yrs(n=9)**	**F-stat**	p_{adj}
Martinsson 2019 [20]	23.14 ± 4.58	19.75 ± 2.65	20.52 ± 3.93	16.86 ± 2.77	9.43	5.9×10^{-5}***
Li 2020 [22]	24.32 ± 4.68	20.80 ± 2.45	21.90 ± 4.53	17.35 ± 3.28	9.57	5.9×10^{-5}***
van Doorn 2021 [21]	23.24 ± 4.54	19.90 ± 2.66	20.73 ± 3.80	17.17 ± 2.89	9.13	5.9×10^{-5}***
Deng 2021 [23]	23.96 ± 5.00	20.16 ± 2.81	21.01 ± 3.87	17.34 ± 2.73	9.93	4.5×10^{-5}***
Rabby 2021 [24]	24.65 ± 4.01	21.73 ± 3.48	22.44 ± 4.51	17.83 ± 3.01	9.40	5.9×10^{-5}***
Lee 2023 [25]	24.42 ± 4.83	20.56 ± 2.75	21.55 ± 4.16	17.68 ± 3.14	10.14	4.0×10^{-5}***
Zhu 2025 [26]	25.46 ± 5.42	21.50 ± 2.99	22.51 ± 4.64	18.44 ± 4.15	8.58	6.4×10^{-5}***
Karagoz 2025 [27]	24.51 ± 4.83	21.16 ± 2.90	22.17 ± 4.38	18.71 ± 3.87	7.06	2.0×10^{-4}***
