# Supplementary material for: Deep learning for blood glucose prediction: Reproducibility challenges and factors affecting differential performance
Source: PLOS Digit Health. 2026 Sep 3;5(9):e0001633. doi: 10.1371/journal.pdig.0001633 (PMC13541153; doi:10.1371/journal.pdig.0001633)
Supplement: Supplementary file 12 [file pdig.0001633.s013.pdf]

S11 Table. Comparison of 30-min prediction RMSE (mg/dL) across insulin delivery modes and corresponding one-way ANOVA results. Subjects pooled across OhioT1DM, DiaTrend, and T1DEXI ($n = 128$). p_{adj} values are Holm–Bonferroni corrected across the family of 8 method tests. Significance: *** $p_{\text{adj}} < 0.001$, ** $p_{\text{adj}} < 0.01$, * $p_{\text{adj}} < 0.05$.

	**30-min BG Pred. RMSE (mg/dL)**				
**Method**	**MDI (n=23)**	**Pump (n=88)**	**Closed Loop (n=17)**	**F-stat**	p_{adj}
Martinsson 2019 [20]	18.26 ± 3.97	22.54 ± 4.17	19.73 ± 3.74	11.68	1.3×10^{-4}***
Li 2020 [22]	19.85 ± 4.82	23.69 ± 4.36	20.41 ± 3.88	9.35	5.0×10^{-4}***
van Doorn 2021 [21]	18.50 ± 3.84	22.69 ± 4.11	19.78 ± 3.71	11.88	1.3×10^{-4}***
Deng 2021 [23]	18.78 ± 3.97	23.26 ± 4.54	20.09 ± 3.72	11.68	1.3×10^{-4}***
Rabby 2021 [24]	21.88 ± 4.78	23.44 ± 4.14	22.72 ± 5.09	1.22	0.299
Lee 2023 [25]	19.36 ± 4.15	23.76 ± 4.43	20.22 ± 3.99	12.14	1.2×10^{-4}***
Zhu 2025 [26]	20.24 ± 5.31	24.81 ± 4.89	20.98 ± 3.83	10.73	2.0×10^{-4}***
Karagoz 2025 [27]	20.49 ± 4.32	23.84 ± 4.57	21.21 ± 4.25	6.48	4.2×10^{-3}**
