# Supplementary material for: Deep learning for blood glucose prediction: Reproducibility challenges and factors affecting differential performance
Source: PLOS Digit Health. 2026 Sep 3;5(9):e0001633. doi: 10.1371/journal.pdig.0001633 (PMC13541153; doi:10.1371/journal.pdig.0001633)
Supplement: Supplementary file 13 [file pdig.0001633.s015.pdf]

February 24, 2026

Dr. Leo Anthony Celi
Editor-in-Chief, PLOS Digital Health

Dr. Kezhi Li
Academic Editor, PLOS Digital Health

Dr. Amara Tariq
Section Editor, PLOS Digital Health

Dear Drs. Celi, Li, and Tariq,

Thank you for the opportunity to revise our manuscript, *"Deep Learning for Blood Glucose Prediction: Reproducibility Challenges and Factors Affecting Differential Performance,"* for consideration at PLOS Digital Health. We have carefully revised the manuscript in accordance with the journal's guidelines and the reviewers' constructive feedback, which has meaningfully strengthened both the clarity and rigor of our work. The updated manuscript and supplementary materials have been prepared in full compliance with the resubmission requirements. Below, we provide a detailed, point-by-point response to the journal's requests and the reviewers' comments.

Thank you for your consideration.

Sincerely,

Baiying Lu
Ph.D. Candidate
Department of Computer Science
Dartmouth College
Email: baiying.lu.gr@dartmouth.edu

Temiloluwa Prioleau, PhD
Assistant Professor
Department of Computer Science
Emory University
Email: tpriole@emory.edu

Journal Requirements:

1. Please ensure that your Ethics Statement is available in its entirety at the beginning of your Methods section, under a subheading 'Ethics Statement'.

In the revised version, we put the Ethics Statement at the beginning of the Methods section to make sure our data and experiments follow the appropriate ethical guidelines, institutional review requirements, and data governance standards.

2. We ask that a manuscript source file is provided at Revision. Please upload your manuscript file as a .doc, .docx, .rtf or .tex.

In this resubmission, we submitted the manuscript_source.tex file that can be used to compile the manuscript.

3. Please upload separate figure files in .tif or .eps format. Also, remove the figures from your manuscript file but keep the legends.

For more information about figure files please see our guidelines:

https://journals.plos.org/digitalhealth/s/figures
https://journals.plos.org/digitalhealth/s/figures#loc-file-requirements

In this resubmission, we also uploaded the figures in .tif format converted by the recommended PLOS's NAAS tool. The figures were removed from the manuscript file, but the figure captions and legends are still kept in the manuscript.

4. We have noticed that you have uploaded Supporting Information files, but you have not included a list of legends. Please add a full list of legends for your Supporting Information files after the references list.

In the revised manuscript, we added legends for all Supporting Information files after the reference list.

If the reviewer comments include a recommendation to cite specific previously published works, please review and evaluate these publications to determine whether they are relevant and should be cited. There is no requirement to cite these works unless the editor has indicated otherwise.

Additional Editor Comments (if provided):

[Note: HTML markup is below. Please do not edit.]

Reviewers' Comments:

Reviewer's Responses to Questions

Comments to the Author

1. Does this manuscript meet PLOS Digital Health's publication criteria? Is the manuscript technically sound, and do the data support the conclusions? The manuscript must describe methodologically and ethically rigorous research with conclusions that are appropriately drawn based on the data presented.

Reviewer #1: Yes

Reviewer #2: Yes

Reviewer #3: Yes

---

2. Has the statistical analysis been performed appropriately and rigorously?

Reviewer #1: Yes

Reviewer #2: Yes

Reviewer #3: Yes

---

3. Have the authors made all data underlying the findings in their manuscript fully available (please refer to the Data Availability Statement at the start of the manuscript PDF file)?

The PLOS Data policy requires authors to make all data underlying the findings described in their manuscript fully available without restriction, with rare exception. The data should be provided as part of the manuscript or its supporting information, or deposited to a public repository. For example, in addition to summary statistics, the data points behind means, medians and variance measures should be available. If there are restrictions on publicly sharing data—e.g. participant privacy or use of data from a third party—those must be specified.

Reviewer #1: Yes

Reviewer #2: Yes

Reviewer #3: No

About this question, we added a statement in the Data availability section (lines 530-532) to further clarify the data availability: For the privacy of study participants, the OhioT1DM, DiaTrend, and T1DEXI datasets are protected via controlled-access managed by the dataset creators/owners. Each dataset can be accessed in accordance with the respective data use agreement.

---

4. Is the manuscript presented in an intelligible fashion and written in standard English?

PLOS Digital Health does not copyedit accepted manuscripts, so the language in submitted articles must be clear, correct, and unambiguous. Any typographical or grammatical errors should be corrected at revision, so please note any specific errors here.

Reviewer #1: Yes

Reviewer #2: Yes

Reviewer #3: Yes

---

5. Review Comments to the Author

Please use the space provided to explain your answers to the questions above. You may also include

additional comments for the author, including concerns about dual publication, research ethics, or publication ethics. (Please upload your review as an attachment if it exceeds 20,000 characters)

Reviewer #1: This research may be applied to both retrospective and prospective glucose-management studies to evaluate data reliability, transparency, and methodological robustness systematically. Incorporating reproducible data practices enhances internal validity by enabling independent verification of findings and reducing the likelihood of analytical bias. In the current landscape of advanced biomedical informatics, deep learning is a practical computational framework for modeling complex, nonlinear glycemic patterns, integrating high-dimensional data, and improving predictive performance.

Thank you.

This is just a thought; when social determinants of health stratify analytical outputs, the model may enable detection of subgroup variation and effect modification, thereby strengthen external validity and advance equitable, evidence-based intervention design.

Thank you. Identifying performance differences across management and demographic subgroups is a part of our analysis. By examining variation in forecasting accuracy across glycemic management levels and demographic factors, our results highlight that individuals with suboptimal glucose control tend to experience higher prediction errors. This finding underscores the importance of ensuring reliable forecasting performance for clinically vulnerable populations.

We also explored performance variation across demographic subgroups (e.g., age, gender, and insulin delivery mode). However, several subgroups were underrepresented and highly imbalanced, limiting the statistical power to draw definitive conclusions regarding demographic effects. As a result, we interpreted these findings cautiously in lines 390-395. In future work, we plan to leverage larger and more diverse datasets to more rigorously evaluate subgroup-specific performance and investigate whether targeted model adaptation or subgroup-specific retraining can improve equitable forecasting performance.

Reviewer #2: Introduction & General:
Need to specify:
Were all the models shortlisted for the study designed for type I diabetes? The S1 file shows otherwise. If not, specify the selection criteria to include how papers based on type-1 or type-2 diabetes were handled for the comparative study.

Thank you for asking this valuable question. To further clarify, S1 presents a comprehensive list of all studies evaluated as part of our literature review on deep learning methods for blood glucose prediction. Among the 8 studies included for reproducibility evaluation, vanDoorn et al, Deng et al, and Lee et al included both type 1 and type 2 patients' data into their model training and evaluation, and they all used the OhioT1DM dataset for model performance evaluation.

In detail, vanDoorn et al. used CGM data from type 2 diabetes patients included in the Maastricht Study for the first step model training, and included type 1 diabetes patients from the OhioT1DM dataset into their training to improve their main model’s adaptation to type 1 diabetes patients. Also, they used the 20% of data from OhioT1DM as an evaluation dataset.

Deng et al. also used CGM data from type 2 diabetes patients from Beth Israel Deaconess Medical Center, but they retrained the model with the union of all the training data of the 12 patients and the testing data of the 6 patients in the 2018 version of OhioT1DM dataset, and evaluated their model on the test part of six patients with type 1 diabetes in 2020 version.

Regarding Lee et al., the model was trained on 226 type 1 patients and 104 type 2 patients. They used the OhioT1DM dataset as an external evaluation dataset.

Our selection criteria are presented in Materials and Methods - Model Selection and Characteristics (from line 157 to 161). In this section, we stated that “we employed the following four criteria: (1) code availability or

detailed model reporting, (2) evaluation on the publicly available OhioT1DM dataset, (3) strong performance for short-term prediction (30 min), and (4) diversity of model architectures.” Consequently, all studies selected for our evaluation had to be evaluated on the publicly available OhioT1DM dataset, independent of whether the respective methods were also evaluated on datasets with type 1 diabetes or type 2 diabetes.

Since the paper mentions that it deals only with Type I cases, it is recommended that this be specified either in the heading or in the abstract. Also, if type-2 is included, there would be models from many other papers to consider. Type 1 diabetes (T1D) and type 2 diabetes (T2D) differ significantly in CGM monitoring patterns and modeling approaches.

Thank you for your feedback. We agree that the models targeting T1D and T2D can be significantly different. In our revised version, we highlight that this study is targeting on T1D glucose forecasting in the abstract: “This study identified key reproducibility challenges associated with current blood glucose prediction methods within type 1 diabetes populations, highlighting the need for better transparency, dataset diversity, standardized evaluation practices, and accessible code to ensure reproducible and reliable models for blood glucose prediction.”

It will be good to mention whether patient separation was considered by the original papers and their implementation.

Thank you for this helpful suggestion. As described in the *Materials and Methods – Data Description* section [Lines 140–143], the OhioT1DM dataset provides a predefined chronological split for each participant, where the first 80% of the time-series data are allocated to training and the remaining 20% to testing. This split is performed within each participant rather than across participants.

In the 8 original studies evaluated in this work, the authors followed this predefined temporal split. In our replication, we adhered to the same protocol to ensure consistency with the original experimental design and to enable fair technical reproducibility evaluation.

Specify the input data that the paper deals with upfront rather than deducing it from the table. Specify the available features in each dataset and then specify the feature characteristic (time series/ tabular data/ etc.)

Thank you for this helpful suggestion. In the revised manuscript, we have expanded the *Materials and Methods – Data Description* section to provide a clearer and more explicit description of the input data, as shown in Lines 103-128. Specifically, we now detail the available features in each dataset and clarify the data characteristics (e.g., longitudinal time-series structure and tabular event-based records). The added content is provided below:

1) **OhioT1DM**: This dataset contains multimodal data collected from wearable devices and manual user logs, including CGM measurements recording at 5-minute intervals, basal and bolus insulin delivery records from insulin pumps, self-reported meal times, meal types, and estimated carbohydrate content, step count data from the Basis Peak sensor band, as well as additional physiological and behavioral information, such as sleep quality, stress levels, and heart rate.

2) **DiaTrend**: The DiaTrend dataset includes data from CGMs and insulin pumps, comprising CGM measurements sampled at 5-minute intervals. However, basal and bolus insulin delivery records are only available for the final 1–2 months of the data collection period.

3) **T1DEXI**: This dataset includes 5-minute–interval CGM time-series data, step count, and physical activity information recorded by the Verily Study Watch, basal and bolus insulin delivery records from insulin pumps or multiple daily injections (MDI), and self-reported meal intake information.

Need to do another proofread to ensure all specifics, including the above-mentioned points about the data, inclusion/exclusion criteria (while selecting a study for comparative analysis), and any implementation details, are explicitly given and need not be inferred by the reader.

Thank you for this important suggestion. We agree that full transparency regarding dataset selection, inclusion/exclusion criteria, and implementation details is essential for a reproducibility study.

In the revised manuscript, we have explicitly clarified the following aspects to ensure that no critical information needs to be inferred by the reader:

1. **Dataset selection and preprocessing criteria.**
   About the dataset, we added further explanation about these three datasets as listed in the previous response (Lines 103-128). Also, we now clearly describe the participant inclusion/exclusion rules for each dataset (e.g., minimum CGM coverage, minimum duration requirements, meal/bolus record availability, and rationale for subset balancing across datasets) in Lines 162-175. We also specify how sampling frequency was standardized (5-minute intervals), how contiguous segments were defined, and how missing data were handled for different model types in Lines 218-227.

2. **Training and evaluation splits.**
   For OhioT1DM, we explicitly follow the dataset release structure (80% training, 20% testing chronologically per participant) (Line 140-143). For DiaTrend and T1DEXI, we clearly state the 5-fold participant-level cross-validation protocol, ensuring that no individual appears in both training and testing folds (Line 272-281).

3. **Model inclusion criteria.**
   We detail the four selection criteria (code availability or sufficient reporting, OhioT1DM usage, 30-minute prediction relevance, architectural diversity) to clarify that model selection was systematic rather than selective (Line 157-161).

4. **Implementation standardization principles.**
   For technical reproducibility, each model was implemented strictly according to the original publication or official code, preserving reported architectures, input features, and preprocessing logic. In the implementation details of each study, we added S2 File and provided further explanation and details of them.
   For statistical reproducibility (Line 241-270), we explicitly standardized:

   - Training dataset (OhioT1DM full training split)
   - Input horizon and sampling interval
   - Evaluation metric (RMSE at 30-minute horizon)
   - Input channel configuration (single-channel CGM vs. multi-channel, as specified)

   For conceptual reproducibility, the input feature configuration followed the same setup as in the statistical reproducibility evaluation. The only two differences from the statistical reproducibility evaluation are that the train/test datasets are DiaTrend and T1DEXI dataset, and the train-test split became a 5-fold cross-validation across participants (Not chronologically 80-20) (Line 272-286).

5. **Per-model configuration details.**
   Because each replicated method has distinct architectural and training specifications, detailed hyperparameters and configuration settings are provided in S2 Table and S2 File. This prevents

overloading the main manuscript while ensuring full reproducibility.

We believe these revisions clarify all dataset-specific decisions, inclusion/exclusion criteria, and implementation settings that could influence reproducibility outcomes. Our goal is to make the experimental pipeline fully transparent and independently replicable without requiring readers to infer unstated assumptions.

Data Description

It would be good to have a short sentence on the relevance of TIR and GV.

Thank you for the suggestion. In the revised version, we added more description about the meaning of TIR and GV in Lines 93-102: In addition, we summarize three clinically recognized metrics to provide further context on glycemic management. The average daily blood glucose reflects the mean of daily blood glucose levels. Time in Range (TIR) is an indicator of glycemic control within the target euglycemic range of 70 - 180 mg/dL and has been associated with a reduced risk of complications. Glycemic variability (GV) captures the degree of glucose fluctuations and is linked to hyper- and hypoglycemia risk and metabolic instability. All three metrics were computed at the daily level for each participant and then averaged across all valid recording days to obtain participant-level summaries.

Are there differences in the sensors used (or their error range) within different studies or datasets? If so, does it affect the comparative study?

The CGM sensors used across the three datasets differ. Participants in the OhioT1DM dataset wore Medtronic Enlite CGM sensors, DiaTrend participants used various CGM systems from Dexcom, Abbott, and Medtronic, and individuals in the T1DEXI study used Dexcom G6 devices. Although these datasets involve different CGM manufacturers and models, all devices comply with ISO 15197:2013 standards and FDA performance requirements. Specifically, they meet accuracy criteria such that ≥95% of measurements fall within ±15 mg/dL (for glucose <100 mg/dL) or ±15% (for glucose ≥100 mg/dL), and >87% fall within ±20%. These sensors are widely used in both clinical practice and routine diabetes management and are considered clinically reliable. Therefore, while minor device-specific variability may exist, we expect its impact on the comparative analysis to be minimal.

We added this explanation to the revised version in Lines 130-137.

The standard can be found at: https://journals.sagepub.com/doi/10.1177/1932296818812062

How is the 80-20 split done in the case of time series in different selected studies and their implementation?

In this study, the 80–20 train–test split was applied only to the OhioT1DM dataset. The original OhioT1DM release already defined this split based on chronological order: for each participant, the first 80% of CGM data were allocated to the training set and the remaining 20% to the test set, following standard time-series forecasting practice. As a result, all selected studies also used the same 80-20 split provided by the original OhioT1DM dataset. This train-test split has been explained in Lines 140-143.

In model selection and characteristics:

What is the reason behind selecting the models evaluated on OhioT1DM only?

We selected models that were evaluated on the OhioT1DM dataset to ensure comparative analysis between the paper-reported results and our reproducibility evaluation. Model evaluation on the same dataset is fundamental for reproducibility evaluation. Furthermore, the OhioT1DM dataset is the most widely used publicly available benchmark in glucose forecasting research. Many prior deep learning studies report results on OhioT1DM, making it a common reference point for methodological comparison. Restricting model

selection to studies that included OhioT1DM evaluation ensured a consistent benchmark for assessing technical, statistical, and conceptual reproducibility. We added this reason into the revised version in Lines 164-169.

Although some of the selected studies also evaluated their models on additional datasets, these were often private datasets (e.g., in Deng et al. and Lee et al.). Because such datasets are publicly available, they do not provide a consistent basis for reproducibility analysis. Therefore, focusing on OhioT1DM ensured transparency, comparability, and methodological rigor in our study.

Unclear on what constitutes diversity of model architectures

Thank you for this clarification request. By "diversity of model architectures," we refer to the inclusion of fundamentally different neural network design paradigms that dominate the glucose forecasting literature. Based on our literature review summarized in S1 File, three primary architectural families are widely adopted: recurrent models (e.g., LSTM-based), convolutional models (CNN-based), and attention-based/Transformer models as we described in Lines 172-175. These paradigms differ substantially in how they model temporal dependencies (sequential recurrence, local convolutional receptive fields, and global self-attention, respectively).

To ensure that our reproducibility evaluation was not limited to a single modeling paradigm, we selected representative and high-performing studies from each of these three architectural categories. This approach allowed us to assess whether reproducibility challenges were architecture-specific or consistent across fundamentally different model designs.

Were any of the original models developed specifically for a specific time of food or not (fasting/random/post-prandial)? Was the aim of implementation for each model kept the same?

Thank you for this important question. All included models were originally developed for short-term continuous blood glucose forecasting (e.g., 30- or 60-minute prediction horizons). None of the models was specifically designed for a particular physiological state or time condition, such as fasting, post-prandial, or random time. Instead, they were trained to predict future glucose values continuously based on recent historical data.

In Results:
One of the major concerns is that participant separation is not at all considered with an 80-20 split, which would result in a good model with less than 20 MSE. (within participant temporal split)

Thank you for this important concern. In our study, the 80–20 temporal split was applied only to the OhioT1DM dataset, following its original release protocol and prior literature conventions. We acknowledge that this within-participant split evaluates temporal generalization rather than cross-subject generalization, but the goal here was for exact replication and rigorous comparison.

To explicitly assess participant-level generalization, we adopted a different strategy for the DiaTrend and T1DEXI datasets. As described in Lines 272-281, we performed 5-fold cross-validation at the participant level, training on four folds and evaluating on the remaining fold, ensuring that test participants were entirely unseen during training. This setup directly evaluates each model's ability to generalize to new individuals and mitigates concerns regarding overly optimistic performance due to within-participant splitting.

Statistical variation is still limited because of no patient separation details

Thank you for this important comment. Similar as the previous question, we acknowledge that a within-participant temporal split (as used in OhioT1DM) primarily evaluates temporal generalization and does not assess inter-patient variability. To address this, we performed participant-level 5-fold cross-validation for the DiaTrend and T1DEXI datasets, where test subjects were entirely unseen during training. This setup explicitly evaluates model generalization across individuals and captures inter-patient statistical variation.

Furthermore, our conceptual reproducibility evaluation extends this assessment by examining model performance across distinct datasets with different cohort characteristics and glycemic profiles. Together, these strategies ensure that both temporal and participant-level variability are systematically evaluated, mitigating concerns about limited statistical variation.

In Conceptual Reproducibility, it will be more meaningful if the developed model were validated instead of retrained on the new datasets, since it gives more information on what concept of the data the model learns from. Which should make it good on new data too.

This is an excellent question, and we appreciate the opportunity to further clarify this point. In response, we conducted additional experiments in which models pretrained on the OhioT1DM dataset were directly evaluated on the DiaTrend and T1DEXI datasets without retraining. The results are reported in S6 Table. In the revised manuscript, we added a short paragraph to describe this finding, reflected in S6 Table in lines 349-352. A more detailed version is as follows.

Comparing S5 (dataset-specific retraining) and S6 (external validation without retraining), we observed only modest performance degradation for most models under cross-dataset evaluation. Excluding Zhu et al. (2025), the average absolute RMSE increase was approximately 0.67 mg/dL on DiaTrend (range: +0.07 to +1.85) and 0.74 mg/dL on T1DEXI (range: +0.38 to +1.26). These relatively small increases suggest that most models retain stable predictive performance despite distributional shifts across datasets.

In contrast, Zhu et al. (2025) showed substantially larger performance degradation, with RMSE increasing by 11.28 mg/dL on DiaTrend and 10.16 mg/dL on T1DEXI, indicating markedly weaker cross-dataset generalization. This pronounced sensitivity suggests that the model may rely more heavily on dataset-specific characteristics learned during pretraining.

Fig 4 needs more interpretation.

Thank you for this suggestion. In our revised version, from Line 364 to 383, we added interpretation based on the results reflected by Fig 4 as follows:

Fig. 4 illustrates the association between blood glucose prediction performance and individual-level diabetes management metrics. Across datasets and models, lower RMSE values were consistently observed in individuals with higher glycemic control (lower mean glucose, higher TIR, lower glycemic variability, and lower HbA1C), whereas higher errors occurred in those with lower glycemic control (higher mean glucose, lower TIR, and higher glycemic variability). The Pearson correlation coefficients ranged from 0.38 to 0.63 (mean: 0.55; $p < 0.001$), indicating moderate-to-strong associations, with up to ~40% of performance variance attributable to management-related factors.

These findings suggest that deep learning models perform substantially better in metabolically stable individuals characterized by smoother and more predictable glucose trajectories. In contrast, individuals with higher glycemic variability and lower TIR exhibit more abrupt fluctuations and nonlinear dynamics, which increase forecasting difficulty. Importantly, this indicates that current models may implicitly favor well-controlled patients, potentially overestimating robustness when evaluated in stable cohorts. The reduced accuracy observed in individuals with poorer glycemic control raises concerns, as these patients may benefit most from reliable glucose prediction. Overall, the results highlight that diabetes management

status is a critical determinant of predictive performance and should be explicitly considered in reproducibility and generalization assessments.

Reviewer #3: Line 41: reference is made to S1 file which isn't part of the document. How would any reader access this information? This is also the case for all references to the Sxx tables and figures. Makes some aspects of the report harder to understand.
Thank you for pointing this out. All supplementary files, tables, and figures (including S1 File and Sxx Tables/Figures) were uploaded and submitted together with the manuscript as supplementary materials. These materials can be accessed and downloaded from the pdf file for reviewing under the supplementary files section.

Fig. 1 caption, references 7 reproducibility criteria however, the figure reports otherwise.

Thank you for pointing out this problem. In our revised version, we changed the “7” in the caption to “8” to match what is shown in Fig. 1.

Table 3: where the code is available the RMSE is much closer to the reported, but still there are some differences. It would be helpful to reflect on this as well in the discussion section. Are there the same number of repeated experiments in both cases; any chance that might have contributed to the difference?

Thank you for this important question. Although full source code was available for several studies (e.g., Martinsson et al. and Deng et al.), minor discrepancies between the originally reported RMSE and our replicated results remain. This is expected in deep neural network models due to inherent stochasticity in training, including random weight initialization, mini-batch sampling order, dropout behavior, and non-deterministic GPU operations. We added this brief explanation to the Result section, Line 302-303.

Regarding repeated experiments, when original studies reported averaged results over multiple runs, we followed the same protocol where possible. However, not all papers clearly specified the number of repetitions or whether the reported value corresponded to the best-performing run or an average across seeds. These reporting differences may also contribute to small discrepancies.

Overall, the observed differences are generally small (within 2% for the two studies with code completely provided: Martinsson et al. and Deng et al.) and fall within a reasonable performance variance range for stochastic deep learning models.

It is not clear from the results if all the demographic effects mentioned - age, gender and insulin delivery mode had enough data individually to help with the the conclusions on demography. Even though there is some reference to S8-10 tables, which are not available in the main report, this should still be made clear or discussed appropriately in the results section.

We thank the reviewer for this insightful question.

Based on the participant distribution shown in S1 Fig, the numbers of participants in each cohort are included in the following tables:

S1 Figure:

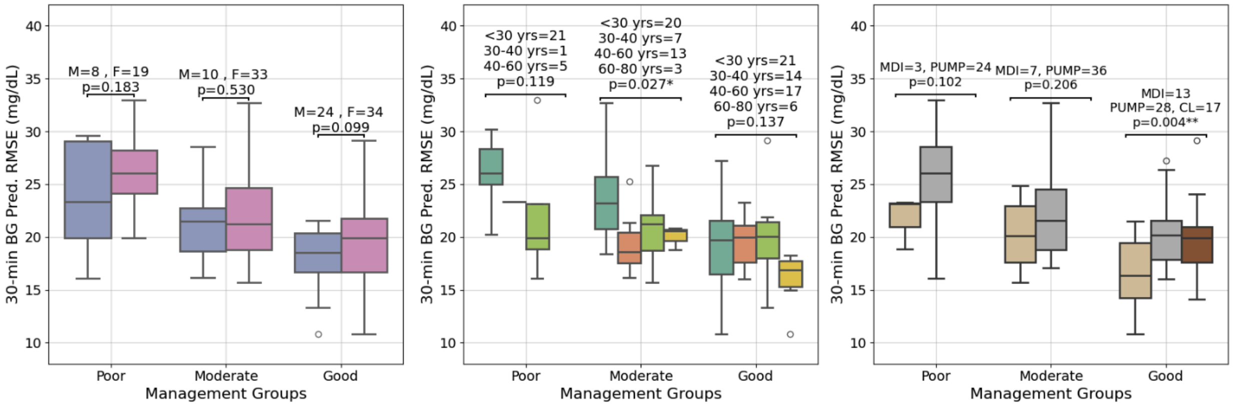

Table A. Participant Distribution by Sex and Glycemic Management Group

Glycemic Management Group	Male (n)	Female (n)	Total (n)
Poor	8	19	27
Moderate	10	33	43
Good	24	34	58
**Total**	**42**	**86**	**128**

Table B. Participant Distribution by Age Cohort and Glycemic Management Group

Glycemic Management Group	<30 yrs	30–40 yrs	40–60 yrs	60–80 yrs	Total
Poor	21	1	5	0	27
Moderate	20	7	13	3	43
Good	21	14	17	6	58
**Total**	**62**	**22**	**35**	**9**	**128**

Table C. Participant Distribution by Insulin Delivery Mode and Glycemic Management Group

Glycemic Management Group	MDI	Pump	Closed-Loop (CL)	Total
Poor	3	24	0	27
Moderate	7	36	0	43
Good	13	28	17	58

**Total**	**23**	**88**	**17**	**128**

As the reviewer correctly noted, several subgroups are imbalanced and, in some cases, under-represented (e.g., only 9 participants aged 60–80 and no closed-loop users in the Poor and Moderate groups). We also further clarified in the revised version in Lines 390 to 394. Given these disparities, we did not further expand correlation analyses between model performance (RMSE) and demographic factors, as such analyses would be statistically underpowered and potentially misleading.

Line 367 possible typo?

Thank you for pointing out this typo. We have corrected this typo in the revised version.

Some reflection on how architectural differences may contribute to the reproducibility would also be interesting.

Thank you for this thoughtful suggestion. We agree that architectural differences may influence reproducibility outcomes. For example, more complex architectures (e.g., Transformer-based models) typically involve larger parameter spaces, additional hyperparameters, and more complex training dynamics, which may increase sensitivity to implementation details and random initialization, complicating the reproduction process. In contrast, simpler architectures such as LSTM- or CNN-based models may exhibit more stable training behavior under similar settings. In addition, especially for the studies that did not fully open their code and did not fully describe their hyperparameter selection, a complicated architecture makes replicating their study/pipeline even more difficult. Given this, we added a brief statement in our discussion section to provide some insights about architectural differences on model reproducibility in Lines 417-419.

Overall, and interesting article but the framework in [12] could be extended to architectural reproducibility.

Thank you! Yes, expanding the reproducibility framework mentioned in [12] to architectural reproducibility is worthwhile. This study now evaluates pipeline-level reproducibility. The reason is that each selected study differs not only in model architecture but also in preprocessing strategies, feature selection, hyperparameter configurations, and evaluation protocols. Because these components are tightly coupled within each original pipeline, it is now still challenging to attribute reproducibility differences solely to architectural design without redesigning and standardizing the entire experimental framework. We have added a brief discussion in lines 417-419 acknowledging that architectural complexity may interact with reproducibility and that a controlled architecture-only comparison would be a valuable direction for future work.
