# Supplementary material for: Deep learning for blood glucose prediction: Reproducibility challenges and factors affecting differential performance
Source: PLOS Digit Health. 2026 Sep 3;5(9):e0001633. doi: 10.1371/journal.pdig.0001633 (PMC13541153; doi:10.1371/journal.pdig.0001633)
Supplement: Supplementary file 14 [file pdig.0001633.s016.pdf]

June 2nd, 2026

Dr. Leo Anthony Celi
Editor-in-Chief, PLOS Digital Health

Dr. Kezhi Li
Academic Editor, PLOS Digital Health

Dear Dr. Celi and Dr. Li,

Thank you for the opportunity to submit a revised version of our manuscript, **"Deep Learning for Blood Glucose Prediction: Reproducibility Challenges and Factors Affecting Differential Performance,"** for consideration at *PLOS Digital Health*. We sincerely appreciate the editor's and reviewers' careful review of our work, as well as their constructive feedback during this minor revision stage.

We have carefully addressed the remaining comments and revised the manuscript accordingly. We believe these updates have further improved the clarity, completeness, and presentation of the paper. The revised manuscript and accompanying materials have been prepared in accordance with the journal's resubmission requirements. Below, we provide a point-by-point response to the remaining comments.

We are grateful for the continued consideration of our manuscript and sincerely hope that the revised version will be suitable for publication in *PLOS Digital Health*.

Thank you again for your time and consideration.

Sincerely,
Baiying Lu
Ph.D. Candidate
Department of Computer Science
Dartmouth College
Email: baiying.lu.gr@dartmouth.edu

Temiloluwa Prioleau, PhD
Assistant Professor
Department of Computer Science
Emory University
Email: tpriole@emory.edu

Journal Requirements:

1. If you are using LaTeX, please ensure that you have provided a single, cohesive .tex file for your LaTeX submission. As stated in the PLOS template, your references should be included in your .tex file (not submitted separately as .bib or .bbl). Please also ensure that you are making any formatting changes to both your .tex file and the PDF of your manuscript. If you have any questions, please contact Latex@plos.org. You can find our LaTeX guidelines here: https://journals.plos.org/digitalhealth/s/latex

Response: In our revised submission, we have included a complete .tex file “manuscript_source.tex” encompassing all sections, figure captions, table captions, and references from both the main text and supplementary materials.

2. If the reviewer comments include a recommendation to cite specific previously published works, please review and evaluate these publications to determine whether they are relevant and should be cited. There is no requirement to cite these works unless the editor has indicated otherwise.

Additional Editor Comments (if provided):
[Note: HTML markup is below. Please do not edit.]

Reviewers' Comments:
Reviewer's Responses to Questions

Comments to the Author

1. If the authors have adequately addressed your comments raised in a previous round of review and you feel that this manuscript is now acceptable for publication, you may indicate that here to bypass the “Comments to the Author” section, enter your conflict of interest statement in the “Confidential to Editor” section, and submit your "Accept" recommendation.

Reviewer #2: All comments have been addressed

Reviewer #3: All comments have been addressed

Reviewer #4: (No Response)

Reviewer #5: All comments have been addressed

---

2. Does this manuscript meet PLOS Digital Health’s publication criteria? Is the manuscript technically sound, and do the data support the conclusions? The manuscript must describe methodologically and ethically rigorous research with conclusions that are appropriately drawn based on the data presented.

Reviewer #2: Yes

Reviewer #3: Yes

Reviewer #4: Yes

Reviewer #5: Yes

---

3. Has the statistical analysis been performed appropriately and rigorously?

Reviewer #2: Yes

Reviewer #3: Yes

Reviewer #4: Yes

Reviewer #5: Yes

---

4. Have the authors made all data underlying the findings in their manuscript fully available (please refer to the Data Availability Statement at the start of the manuscript PDF file)?

The PLOS Data policy requires authors to make all data underlying the findings described in their manuscript fully available without restriction, with rare exception. The data should be provided as part of the manuscript or its supporting information, or deposited to a public repository. For example, in addition to summary statistics, the data points behind means, medians and variance measures should be available. If there are restrictions on publicly sharing data—e.g. participant privacy or use of data from a third party—those must be specified.

Reviewer #2: Yes

Reviewer #3: Yes

Reviewer #4: Yes

Reviewer #5: Yes

---

5. Is the manuscript presented in an intelligible fashion and written in standard English?

PLOS Digital Health does not copyedit accepted manuscripts, so the language in submitted articles must be clear, correct, and unambiguous. Any typographical or grammatical errors should be corrected at revision, so please note any specific errors here.

Reviewer #2: Yes

Reviewer #3: Yes

Reviewer #4: Yes

Reviewer #5: Yes

---

6. Review Comments to the Author

Please use the space provided to explain your answers to the questions above. You may also include additional comments for the author, including concerns about dual publication, research ethics, or publication ethics. (Please upload your review as an attachment if it exceeds 20,000 characters)

Reviewer #2: Thank you for addressing all the comments.

Reviewer #3: (No Response)

Reviewer #4:

This study aims to identify key reproducibility challenges in deep learning (DL) algorithms for blood glucose (BG) forecasting in type 1 diabetes (T1D). To this end, the authors reviewed 67 recent papers on DL-based BG forecasting and rigorously reimplemented and evaluated eight representative algorithms. Specifically, they assessed reproducibility under three paradigms: technical, statistical, and conceptual reproducibility. Technical reproducibility examines whether the predictive performance reported in the original studies can be replicated under the same experimental conditions (i.e., identical data, preprocessing, model architecture, and evaluation pipeline). Statistical reproducibility evaluates whether models achieve statistically comparable performance under a slightly different but standardized experimental setting. This unified framework harmonizes inputs and evaluation protocols, enabling fair comparisons across models. Finally, conceptual reproducibility assesses the ability of models to generalize to different T1D cohorts.
The key contributions of this study are threefold: (1) the identification of major challenges that hinder consistent replication, (2) a systematic comparison of state-of-the-art models under a unified and standardized experimental framework, and (3) an analysis of factors influencing performance variability, highlighting glucose management as the most significant determinant of model performance.
Overall, I find this study to be well written, highly relevant, and worthy of publication, as it addresses a critical gap in the field. However, I have a few minor suggestions for improvement.

Response: We sincerely thank the reviewer for the careful and thoughtful evaluation of our manuscript. We are grateful for the reviewer’s positive assessment of the study and for recognizing its relevance and contribution to addressing reproducibility challenges in deep learning-based blood glucose forecasting. We appreciate the reviewer’s constructive minor suggestions and have addressed them in detail below.

Minor Revisions
1. The authors performed several statistical analyses involving multiple tests (e.g., Table S7–S11, S1 Fig. 1, Fig. 4, Fig. 3D–E, and Fig. 2), but it is not clear whether corrections for multiple comparisons were applied. For example, in Tables S7–S11, separate t-tests/ANOVAs are conducted for each model, which would typically require adjustment for multiple testing. Similarly, in S1 Fig. 1, nine statistical tests are performed across the three panels. I therefore recommend applying an appropriate multiple-comparisons correction (e.g., Bonferroni, Holm–Bonferroni, or FDR control) to ensure that the reported significance levels are robust and to reduce the risk of false positive inflation.

Response: We thank the reviewer for this helpful recommendation. In the revised manuscript and supplementary materials, we applied Holm–Bonferroni correction to the relevant families of statistical tests reported in Tables S7–S11, S1 Fig. 1, Fig. 2, Fig. 3D–E, and Fig. 4. The reported p-values have been updated to Holm–Bonferroni-adjusted p-values where applicable, and the corresponding figure and table captions have been revised to explicitly state the correction procedure.

Overall, the adjusted results remain largely consistent with the findings reported in the original submission. For example, in Fig. 2, the distributions of daily average glucose level and daily average TIR ratio remain significantly different across the three datasets after correction. In Fig. 3D–E, the main conclusions are unchanged; the only change is that the significance level for the OhioT1DM–DiaTrend performance difference for Zhu et al. (2025) and Karagoz et al. (2025) changed from $p < 0.001$ to $p_{adj} < 0.01$, remaining significant. In Fig. 4, the correlation analyses remain consistent with the original findings after adjustment.

The only major difference after correction appears in Table S9: the previously observed performance differences between gender groups are no longer statistically significant after Holm–Bonferroni adjustment, with adjusted p-values greater than 0.05. This finding is consistent with our broader observation that sex/gender was not a major determinant of forecasting performance compared with glucose management-related factors (in Line 395-397). We also removed gender from the sentence in our Discussion section, “While the correlation analysis reported that demographic factors such as (gender), age,

and insulin delivery mode are highly relevant to prediction accuracy, …", only keeping the age and insulin delivery mode.

2. When introducing the standard $RMSE_{30}$ and the customized RMSEseq used by Karagoz et al., I suggest clarifying that $RMSE_{30}$ represents the standard approach according to best-practice guidelines (DOI: 10.1109/RBME.2023.3331297) and recent literature reviews (DOI: 10.1109/JBHI.2025.3630214). This clarification would also strengthen the rationale for selecting $RMSE_{30}$ over alternative metrics in the statistical reproducibility assessment.

Response: We thank the reviewer for this helpful suggestion. In Lines 213–219 of the revised manuscript, we clarified that RMSE30 represents the standard 30-minute prediction-horizon evaluation metric commonly used in blood glucose prediction studies and adopted it as the primary evaluation metric in our statistical reproducibility assessment. We also added the recommended best-practice guideline and recent literature review as references to strengthen the rationale for using RMSE30 over alternative metrics.

3. I recommend adding a note explaining that the apparently improved performance reported by Karagoz et al. is primarily driven by differences in RMSE calculation. Consequently, these results are not directly comparable to other studies without a proper statistical reproducibility evaluation.

Response: We thank the reviewer for this helpful suggestion. In the revised manuscript, we added a clarification that the RMSE calculation used by Karagoz et al. (2025), which averages errors over the entire prediction sequence rather than evaluating the fixed 30-minute prediction horizon, can lead to lower reported RMSE (in Line 213-214). We agree and have awared that these reported results are not directly comparable to those from studies using the standard RMSE30 metric.

To avoid misleading comparisons, in the Technical Reproducibility section, we just presented the originally reported results but did not directly compare Karagoz et al.'s reported RMSE with results from other methods. Direct comparisons are made only in the Statistical Reproducibility section, where the input settings and evaluation metrics are standardized across methods, including the use of RMSE30 as the primary evaluation metric.

4. Recently, a systematic review on deep learning algorithms for BG forecasting (DOI: 10.1109/JBHI.2025.3630214) highlighted the challenges associated with cross-study performance comparison and emphasized the need for standardized evaluation practices. This reference could be used to further support the motivation and discussion of your study.

Response: We thank the reviewer for pointing us to this relevant systematic review. In Line 20 of the revised manuscript, we have added this reference to further support the motivation of our study, particularly the need for standardized evaluation practices and the challenges associated with comparing reported performance across blood glucose forecasting studies.

5. The readability of the manuscript would benefit from briefly defining technical, statistical, and conceptual reproducibility already in the introduction. Without this clarification, these terms may appear abstract to the reader at this stage.

Response: We thank the reviewer for this helpful suggestion. In the Introduction section Line 43-47 of the manuscript, we have briefly defined technical, statistical, and conceptual reproducibility following McDermott et al. Specifically, we define these concepts as: (1) technical reproducibility, which asks whether results can be reproduced under technically identical conditions; (2) statistical reproducibility, which asks whether results can be reproduced under statistically comparable conditions; and (3) conceptual reproducibility, or replicability, which asks whether results can be reproduced under conceptually similar conditions. A more detailed description of how these concepts are operationalized and applied in our study is provided later in the manuscript (Line 236-241, Line 248-253, Line 278-281).

6. Line 98: Glycemic variability (GV) is a broad term and does not refer to a specific metric. It appears that the authors intend GV to represent the coefficient of variation (CV) of blood glucose (BG); this should be explicitly clarified.

Response: We thank the reviewer for this helpful suggestion. We agree that glycemic variability is a broad concept and should not be presented as a single metric without clarification. In the revised manuscript Line 100-102, we clarified in the Materials and Methods—Data Description section that glycemic variability in this study was quantified using the coefficient of variation (CV) of blood glucose values. We also added the corresponding definition, specifying that CV is calculated as the standard deviation of glucose values divided by the mean glucose value and expressed as a percentage.

7. Line 138: The authors state: "To account for differences across the three datasets, an inclusion criterion was applied to ensure consistency and analytical validity." However, it appears that different inclusion criteria are applied to each dataset. This should be clarified explicitly in the text.

Response: We thank the reviewer for pointing out this ambiguity. We agree that the original wording could suggest that a single identical inclusion criterion was applied across all three datasets. In the revised manuscript (Line 140-157), we clarify that the inclusion criteria were dataset-specific, reflecting differences in dataset size, recording duration, sampling frequency, and annotation completeness. However, the shared objective of these criteria was to ensure sufficient CGM continuity, availability of meal and bolus annotations for event-informed analyses, comparable analytic sample sizes, and harmonized 5-minute CGM resolution across datasets.

8. Table 2: Please clarify in caption or notes that the algorithm from Lee et al. could not be reproduced due to the use of a private dataset.

Response: We thank the reviewer for this helpful suggestion. In Table 3 of the revised manuscript, we added Note 4 to clarify that the algorithm from Lee et al. could not be evaluated for technical reproducibility because the model was trained using a private dataset that was not publicly accessible. Therefore, the original training conditions could not be reproduced.

9. Table S4: I recommend removing the phrase "per participant," as this would typically suggest a detailed table reporting RMSE values for each individual participant.

Response: We thank the reviewer for this helpful comment. We agree that the phrase "per participant" did not accurately reflect the content of Table S4, as the table reports overall performance rather than individual participant-level RMSE values. In the revised supplementary materials, we removed this phrase and replaced it with "Overall."

10. Table S5: I suggest removing the term "RMSE" after "DiaTrend:" and "T1DEXI:", as it may imply that all reported columns refer only to RMSE, whereas they also include MAE.

Response: We thank the reviewer for pointing out this detail. We agree that including "RMSE" after "DiaTrend" and "T1DEXI" could incorrectly suggest that all reported columns refer only to RMSE, although the table also includes MAE. In the revised S5 Table, we removed "RMSE" from these labels to avoid ambiguity.

11. Table S7: I suggest also reporting the mean differences shown in Fig. 3 within the table for completeness.

Response: We thank the reviewer for this helpful suggestion. In the revised Table S7, we added the mean differences shown in Fig. 3 to provide a more complete summary of the dataset-level performance comparisons.

12. Table S8: I suggest indicating whether the reported correlation coefficients are statistically significant to improve interpretability.

Response: We thank the suggestion from the reviewer. In the updated Table S8, we indicated in the caption that all of the correlation coefficients are highly significant ($p<0.001$).

13. Table S9: The number of male and female should be reported as in Table S10 for age groups.

Response: We thank the suggestion from the reviewer. In revised Table S9, we updated the number of male and female in the caption and in the table.

14. S1 Figure: I suggest
a. Clarifying how the management groups were defined in the figure caption, as this is currently unclear.
b. Clarify the statistical tests you performed.
c. Colouring the text indicating the groups to match the corresponding boxplot colors.

Response: We thank the reviewer for these helpful suggestions. We revised S1 Fig. and its caption to address all three points.

a. First, we clarified the definition of the glucose management groups in the figure caption. Specifically, glucose management groups were defined based on each participant's average daily time-in-range (TIR) ratio: participants with TIR ratio < 0.5 were assigned to the poor management group, those with 0.5 ≤ TIR ratio < 0.7 were assigned to the moderate management group, and those with TIR ratio ≥ 0.7 were assigned to the good management group.
b. Second, we clarified the statistical tests used in the figure caption. Within each management group, we compared 30-minute prediction RMSE by sex using independent two-sample t-tests. We also compared RMSE across age categories and insulin delivery modes using one-way ANOVA. For each family of subgroup tests, raw p-values were adjusted using the Holm–Bonferroni correction to account for multiple comparisons.
c. Third, in the updated S1 Fig., we added legends to each subplot to clarify the mapping between each subgroup and the corresponding boxplot color.

Typos
1. In the first sentence of the Author Summary, there is a typo: "food, activity, insulin use, and more after (alter?) their blood glucose levels."

Response: The sentence in the submitted manuscript reads: "Managing type 1 diabetes requires individuals to frequently monitor their blood glucose levels and constantly consider how decisions related to food, activity, insulin use, and more affect their blood glucose levels." We confirmed that the sentence uses "affect," not "after" or "alter."

2. Line 5: "management of blood glucose (BG) levels to maintain (to) a healthy standard of living"

Response: We corrected the sentence by removing the extra "to" in "to maintain a healthy standard of living."
3. Line 293: "demographic factors (contributeto) variability"

Response: We checked the sentence and confirmed that it reads: "We further analyzed how individual diabetes management factors and demographic factors contribute to variability in model performance across

datasets, identifying the fundamental reason that strongly affects prediction accuracy.” The phrase “contribute to” appears correctly separated in the submitted manuscript.

4. Line 329: “we can observe (varyinglevels) of increased error”.

Response: We checked the sentence and confirmed that “varying levels” appears as two separate words in the submitted manuscript. This may have resulted from formatting or rendering during review.

5. Line 463: “differential (performanceobserved) across”.

Response: We checked the sentence and confirmed that “performance observed” appears as two separate words in the submitted manuscript. This may have resulted from formatting or rendering during review.

6. Line 478: “(DatasetTransparency) and Reporting Standards:”

Response: We checked the heading and confirmed that “Dataset Transparency” appears as two separate words in the submitted manuscript. This may have resulted from formatting or rendering during review.

Reviewer #5: The manuscript addresses an important and often under-discussed reproducibility issue in blood glucose prediction research. In practice, many published works in this area lack sufficient implementation details or methodological clarity, which makes reproducing reported performance difficult. Also, the study provides a valuable contribution by investigating the relationship between model performance and glycemic management, which may help explain variability in reported results across studies and patient cohorts.

A particular strength of the work is that the authors examine reproducibility from multiple perspectives, including technical, statistical, and conceptual reproducibility. This distinction is presented clearly throughout the manuscript and provides a clearcut framework for analysing reproducibility in blood glucose prediction research.

The methodology appears technically sound, and the conclusions are appropriately supported by the presented data. The manuscript also highlights inter-patient variability in prediction performance, and the use of five-fold cross-validation is therefore appropriate for evaluating model robustness across different patient populations.

In addition, the authors have made efforts to support reproducibility by providing the implementation code through a public GitHub repository. The manuscript also clearly states how the dataset used in the study can be accessed, including any applicable restrictions. Overall, the manuscript is clearly written and presented in an intelligible manner.

Response: We sincerely thank Reviewer #5 for the thoughtful and positive evaluation of our manuscript. We are grateful that the reviewer recognized the importance of the reproducibility problem in blood glucose prediction research, the value of our technical/statistical/conceptual reproducibility framework, and our efforts to improve transparency through public code release and clear dataset-access documentation.
