# Supplementary material for: Deep learning for blood glucose prediction: Reproducibility challenges and factors affecting differential performance
Source: PLOS Digit Health. 2026 Sep 3;5(9):e0001633. doi: 10.1371/journal.pdig.0001633 (PMC13541153; doi:10.1371/journal.pdig.0001633)
Supplement: Peer review history

# Peer review: Decision Letter 0

**DOI:** 10.1371/journal.pdig.0001633.r001

**Authors:** Kezhi Li, Amara Tariq

29 Jan 2026

Response to Reviewers Revised Manuscript with Track Changes Manuscript **Journal Requirements:**

1. Please ensure that your Ethics Statement is available in its entirety at the beginning of your Methods section, under a subheading 'Ethics Statement'.

2. We ask that a manuscript source file is provided at Revision. Please upload your manuscript file as a .doc, .docx, .rtf or .tex.

3. Please upload separate figure files in .tif or .eps format. Also, remove the figures from your manuscript file but keep the legends.

For more information about figure files please see our guidelines:

https://journals.plos.org/digitalhealth/s/figures

https://journals.plos.org/digitalhealth/s/figures#loc-file-requirements

4. We have noticed that you have uploaded Supporting Information files, but you have not included a list of legends. Please add a full list of legends for your Supporting Information files after the references list.

**Additional Editor Comments (if provided):** **Reviewers' Comments:**

**Comments to the Author**

1. Does this manuscript meet PLOS Digital Health’s publication criteria?

Reviewer #1: Yes

Reviewer #2: Yes

Reviewer #3: Yes

**********

2. Has the statistical analysis been performed appropriately and rigorously?

Reviewer #1: Yes

Reviewer #2: Yes

Reviewer #3: Yes

**********

3. Have the authors made all data underlying the findings in their manuscript fully available (please refer to the Data Availability Statement at the start of the manuscript PDF file)?

The PLOS Data policy

Reviewer #1: Yes

Reviewer #2: Yes

Reviewer #3: No

**********

4. Is the manuscript presented in an intelligible fashion and written in standard English?

Reviewer #1: Yes

Reviewer #2: Yes

Reviewer #3: Yes

**********

Reviewer #1: This research may be applied to both retrospective and prospective glucose-management studies to evaluate data reliability, transparency, and methodological robustness systematically. Incorporating reproducible data practices enhances internal validity by enabling independent verification of findings and reducing the likelihood of analytical bias. In the current landscape of advanced biomedical informatics, deep learning is a practical computational framework for modeling complex, nonlinear glycemic patterns, integrating high-dimensional data, and improving predictive performance.

This is just a thought; when social determinants of health stratify analytical outputs, the model may enable detection of subgroup variation and effect modification, thereby strengthen external validity and advance equitable, evidence-based intervention design.

Reviewer #2: Introduction & General:

Need to specify:

Were all the models shortlisted for the study designed for type I diabetes? The S1 fille shows otherwise. If not, specify the selection criteria to include how papers based on type-1 or type-2 diabetes were handled for the comparative study.

Since the paper mentions that it deals only with Type I cases, it is recommended that this be specified either in the heading or in the abstract. Also, if type-2 is included, there would be models from many other papers to consider. Type 1 diabetes (T1D) and type 2 diabetes (T2D) differ significantly in CGM monitoring patterns and modeling approaches.

It will be good to mention whether patient separation was considered by the original papers and their implementation.

Specify the input data that the paper deals with upfront rather than deducing it from the table. Specify the available features in each dataset and then specify the feature characteristic (time series/ tabular data/ etc.)

Need to do another proofread to ensure all specifics, including the above-mentioned points about the data, inclusion/exclusion criteria (while selecting a study for comparative analysis), and any implementation details, are explicitly given and need not be inferred by the reader.

Data Description

It would be good to have a short sentence on the relevance of TIR and GV.

Are there differences in the sensors used (or their error range) within different studies or datasets? If so, does it affect the comparative study?

How is the 80-20 split done in the case of time series in different selected studies and their implementation?

In model selection and characteristics:

What is the reason behind selecting the models evaluated on OhioT1DM only?

Unclear on what constitutes diversity of model architectures

Were any of the original models developed specifically for a specific time of food or not (fasting/random/post-prandial)? Was the aim of implementation for each model kept the same?

In Results:

One of the major concerns is that participant separation is not at all considered with a 80-20 split, which would result in a good model with less than 20 MSE. (within participant temporal split)

Statistical variation is still limited because of no patient separation details

In Conceptual Reproducibility, It will be more meaningful if the developed model were validated instead of retrained on the new datasets, since it gives more information on what concept of the data the model learns from? Which should make it good on new data too.

Fig 4 needs more interpretation.

Reviewer #3: Line 41: reference is made to S1 file which isn't part of the document. How would any reader access this information? This is also the case for all references to the Sxx tables and figures. Makes some aspects of the report harder to understand.

Fig. 1 caption, references 7 reproducibility criteria however the figure reports otherwise.

Table 3: where the code is available the RMSE is much closer to the reported, but still there are some differences. It would be helpful to reflect on this as well in the discussion section. Are there the same number of repeated experiments in both cases; any chance that might have contributed to the difference?

It is not clear from the results if all the demographic effects mentioned - age, gender and insulin delivery mode had enough data individually to help with the the conclusions on demography. Even though there is some reference to S8-10 tables, which are not available in the main report, this should still be made clear or discussed appropriately in the results section.

Line 367 possible typo?

Some reflection on how architectural differences may contribute to the reproducibility would also be interesting.

Overall, and interesting article but the framework in [12] could be extended to architectural reproducibility.

**********

what does this mean?). If published, this will include your full peer review and any attached files.

**Do you want your identity to be public for this peer review?**  If you choose “no”, your identity will remain anonymous but your review may still be made public.

For information about this choice, including consent withdrawal, please see our Privacy Policy

Reviewer #1: No

Reviewer #2: **Yes:**  Rahul Krishnan Pathinarupothi

Reviewer #3: No

**********

**Figure resubmission:**

**Reproducibility:**

To enhance the reproducibility of your results, we recommend that authors of applicable studies deposit laboratory protocols in protocols.io, where a protocol can be assigned its own identifier (DOI) such that it can be cited independently in the future. Additionally, PLOS ONE offers an option to publish peer-reviewed clinical study protocols. Read more information on sharing protocols at https://plos.org/protocols?utm_medium=editorial-email&utm_source=authorletters&utm_campaign=protocols

# Author comment: Author response to Decision Letter 1

**DOI:** 10.1371/journal.pdig.0001633.r002

17 Mar 2026

Attachment Submitted filename: Response to Reviewers.pdf

# Peer review: Decision Letter 1

**DOI:** 10.1371/journal.pdig.0001633.r003

**Authors:** Kezhi Li, Amara Tariq

25 May 2026

Response to Reviewers Revised Manuscript with Track Changes Manuscript **Journal Requirements:**

1. If you are using LaTeX, please ensure that you have provided a single, cohesive .tex file for your LaTeX submission. As stated in the PLOS template, your references should be included in your .tex file (not submitted separately as .bib or .bbl). Please also ensure that you are making any formatting changes to both your .tex file and the PDF of your manuscript. If you have any questions, please contact Latex@plos.org. You can find our LaTeX guidelines here: https://journals.plos.org/digitalhealth/s/latex

2. If the reviewer comments include a recommendation to cite specific previously published works, please review and evaluate these publications to determine whether they are relevant and should be cited. There is no requirement to cite these works unless the editor has indicated otherwise.

**Additional Editor Comments (if provided):** **Reviewers' Comments:**

**Comments to the Author**

Reviewer #2: All comments have been addressed

Reviewer #3: All comments have been addressed

Reviewer #4: (No Response)

Reviewer #5: All comments have been addressed

**********

publication criteria?

Reviewer #2: Yes

Reviewer #3: Yes

Reviewer #4: Yes

Reviewer #5: Yes

**********

3. Has the statistical analysis been performed appropriately and rigorously?

Reviewer #2: Yes

Reviewer #3: Yes

Reviewer #4: Yes

Reviewer #5: Yes

**********

4. Have the authors made all data underlying the findings in their manuscript fully available (please refer to the Data Availability Statement at the start of the manuscript PDF file)?

The PLOS Data policy

Reviewer #2: Yes

Reviewer #3: Yes

Reviewer #4: Yes

Reviewer #5: Yes

**********

5. Is the manuscript presented in an intelligible fashion and written in standard English?

Reviewer #2: Yes

Reviewer #3: Yes

Reviewer #4: Yes

Reviewer #5: Yes

**********

Reviewer #2: Thank you for addressing all the comments.

Reviewer #3: (No Response)

Reviewer #4: This study aims to identify key reproducibility challenges in deep learning (DL) algorithms for blood glucose (BG) forecasting in type 1 diabetes (T1D). To this end, the authors reviewed 67 recent papers on DL-based BG forecasting and rigorously reimplemented and evaluated eight representative algorithms. Specifically, they assessed reproducibility under three paradigms: technical, statistical, and conceptual reproducibility.

Technical reproducibility examines whether the predictive performance reported in the original studies can be replicated under the same experimental conditions (i.e., identical data, preprocessing, model architecture, and evaluation pipeline). Statistical reproducibility evaluates whether models achieve statistically comparable performance under a slightly different but standardized experimental setting. This unified framework harmonizes inputs and evaluation protocols, enabling fair comparisons across models. Finally, conceptual reproducibility assesses the ability of models to generalize to different T1D cohorts.

The key contributions of this study are threefold: (1) the identification of major challenges that hinder consistent replication, (2) a systematic comparison of state-of-the-art models under a unified and standardized experimental framework, and (3) an analysis of factors influencing performance variability, highlighting glucose management as the most significant determinant of model performance.

Overall, I find this study to be well written, highly relevant, and worthy of publication, as it addresses a critical gap in the field. However, I have a few minor suggestions for improvement.

Minor Revisions

1. The authors performed several statistical analyses involving multiple tests (e.g., Table S7–S11, S1 Fig. 1, Fig. 4, Fig. 3D–E, and Fig. 2), but it is not clear whether corrections for multiple comparisons were applied. For example, in Tables S7–S11, separate t-tests/ANOVAs are conducted for each model, which would typically require adjustment for multiple testing. Similarly, in S1 Fig. 1, nine statistical tests are performed across the three panels. I therefore recommend applying an appropriate multiple-comparisons correction (e.g., Bonferroni, Holm–Bonferroni, or FDR control) to ensure that the reported significance levels are robust and to reduce the risk of false positive inflation.

2. When introducing the standard RMSE₃₀ and the customized RMSEseq used by Karagoz et al., I suggest clarifying that RMSE₃₀ represents the standard approach according to best-practice guidelines (DOI: 10.1109/RBME.2023.3331297) and recent literature reviews (DOI: 10.1109/JBHI.2025.3630214). This clarification would also strengthen the rationale for selecting RMSE₃₀ over alternative metrics in the statistical reproducibility assessment.

3. I recommend adding a note explaining that the apparently improved performance reported by Karagoz et al. is primarily driven by differences in RMSE calculation. Consequently, these results are not directly comparable to other studies without a proper statistical reproducibility evaluation.

4. Recently, a systematic review on deep learning algorithms for BG forecasting (DOI: 10.1109/JBHI.2025.3630214) highlighted the challenges associated with cross-study performance comparison and emphasizes the need for standardized evaluation practices. This reference could be used to further support the motivation and discussion of your study.

5. The readability of the manuscript would benefit from briefly defining technical, statistical, and conceptual reproducibility already in the introduction. Without this clarification, these terms may appear abstract to the reader at this stage.

6. Line 98: Glycemic variability (GV) is a broad term and does not refer to a specific metric. It appears that the authors intend GV to represent the coefficient of variation (CV) of blood glucose (BG); this should be explicitly clarified.

7. Line 138: The authors state: “To account for differences across the three datasets, an inclusion criterion was applied to ensure consistency and analytical validity.” However, it appears that different inclusion criteria are applied to each dataset. This should be clarified explicitly in the text.

8. Table 2: Please clarify in caption or notes that the algorithm from Lee et al. could not be reproduced due to the use of a private dataset.

9. Table S4: I recommend removing the phrase “per participant,” as this would typically suggest a detailed table reporting RMSE values for each individual participant.

10. Table S5: I suggest removing the term “RMSE” after “DiaTrend:” and “T1DEXI:”, as it may imply that all reported columns refer only to RMSE, whereas they also include MAE.

11. Table S7: I suggest also reporting the mean differences shown in Fig. 3 within the table for completeness.

12. Table S8: I suggest indicating whether the reported correlation coefficients are statistically significant to improve interpretability.

13. Table S9: The number of male and female should be reported as in Table S10 for age groups.

14. S1 Figure: I suggest

a. Clarifying how the management groups were defined in the figure caption, as this is currently unclear.

b. Clarify the statistical tests you performed.

c. Colouring the text indicating the groups to match the corresponding boxplot colors.

Typos

1. In the first sentence of the Author Summary, there is a typo: “food, activity, insulin use, and more after (alter?) their blood glucose levels.”

2. Line 5: “management of blood glucose (BG) levels to maintain (to) a healthy standard of living”

3. Line 293: “demographic factors (contributeto) variability”

4. Line 329: “we can observe (varyinglevels) of increased error”

5. Line 463: “differential (performanceobserved) across”

6. Line 478: “(DatasetTransparency) and Reporting Standards:”

Reviewer #5: The manuscript addresses an important and often under-discussed reproducibility issue in blood glucose prediction research. In practice, many published works in this area lack sufficient implementation details or methodological clarity, which makes reproducing reported performance difficult. Also, the study provides a valuable contribution by investigating the relationship between model performance and glycemic management, which may help explain variability in reported results across studies and patient cohorts.

A particular strength of the work is that the authors examine reproducibility from multiple perspectives, including technical, statistical, and conceptual reproducibility. This distinction is presented clearly throughout the manuscript and provides a clearcut framework for analysing reproducibility in blood glucose prediction research.

The methodology appears technically sound, and the conclusions are appropriately supported by the presented data. The manuscript also highlights inter-patient variability in prediction performance, and the use of five-fold cross-validation is therefore appropriate for evaluating model robustness across different patient populations.

In addition, the authors have made efforts to support reproducibility by providing the implementation code through a public GitHub repository. The manuscript also clearly states how the dataset used in the study can be accessed, including any applicable restrictions. Overall, the manuscript is clearly written and presented in an intelligible manner.

**********

what does this mean?). If published, this will include your full peer review and any attached files.

**Do you want your identity to be public for this peer review?**  If you choose “no”, your identity will remain anonymous but your review may still be made public.

For information about this choice, including consent withdrawal, please see our Privacy Policy

Reviewer #2: **Yes:**  Rahul Krishnan Pathinarupothi

Reviewer #3: No

Reviewer #4: No

Reviewer #5: **Yes:**  Luyuan Qi

**********

**Figure resubmission:**

**Reproducibility:**

To enhance the reproducibility of your results, we recommend that authors of applicable studies deposit laboratory protocols in protocols.io, where a protocol can be assigned its own identifier (DOI) such that it can be cited independently in the future. Additionally, PLOS ONE offers an option to publish peer-reviewed clinical study protocols. Read more information on sharing protocols at https://plos.org/protocols?utm_medium=editorial-email&utm_source=authorletters&utm_campaign=protocols

# Author comment: Author response to Decision Letter 2

**DOI:** 10.1371/journal.pdig.0001633.r004

4 Jun 2026

Attachment Submitted filename: Response_to_Reviewers_auresp_2.pdf

# Peer review: Decision Letter 2

**DOI:** 10.1371/journal.pdig.0001633.r005

**Authors:** Amara Tariq

19 Jul 2026

Deep learning for blood glucose prediction: reproducibility challenges and factors affecting differential performance

PDIG-D-25-01058R2

Dear Ms Lu,

We are pleased to inform you that your manuscript 'Deep learning for blood glucose prediction: reproducibility challenges and factors affecting differential performance' has been provisionally accepted for publication in PLOS Digital Health.

Before your manuscript can be formally accepted you will need to complete some formatting changes, which you will receive in a follow-up email from a member of our team.

Please note that your manuscript will not be scheduled for publication until you have made the required changes, so a swift response is appreciated.

IMPORTANT: The editorial review process is now complete. PLOS will only permit corrections to spelling, formatting or significant scientific errors from this point onwards. Requests for major changes, or any which affect the scientific understanding of your work, will cause delays to the publication date of your manuscript.

If your institution or institutions have a press office, please notify them about your upcoming paper to help maximize its impact. If they'll be preparing press materials, please inform our press team as soon as possible -- no later than 48 hours after receiving the formal acceptance. Your manuscript will remain under strict press embargo until 2 pm Eastern Time on the date of publication. For more information, please contact digitalhealth@plos.org.

Thank you again for supporting Open Access publishing; we are looking forward to publishing your work in PLOS Digital Health.

Best regards,

Amara Tariq

Section Editor

PLOS Digital Health

***********************************************************

**Additional Editor Comments (if provided):**

**Reviewer Comments (if any, and for reference):**

Reviewer's Responses to Questions

**Comments to the Author**

Reviewer #4: All comments have been addressed

**********

publication criteria?

Reviewer #4: Yes

**********

3. Has the statistical analysis been performed appropriately and rigorously?

Reviewer #4: Yes

**********

4. Have the authors made all data underlying the findings in their manuscript fully available (please refer to the Data Availability Statement at the start of the manuscript PDF file)?

The PLOS Data policy

Reviewer #4: Yes

**********

5. Is the manuscript presented in an intelligible fashion and written in standard English?

Reviewer #4: Yes

**********

Reviewer #4: Thank you for carefully addressing all the comments. The manuscript has improved considerably and is now suitable for publication.

I also apologize for pointing out a few apparent typographical errors that were in fact rendering issues rather than errors in the manuscript itself.

**********

what does this mean?). If published, this will include your full peer review and any attached files.

**Do you want your identity to be public for this peer review?**  If you choose “no”, your identity will remain anonymous but your review may still be made public.

For information about this choice, including consent withdrawal, please see our Privacy Policy

Reviewer #4: No

**********
